# Solar electricity and fuel production with perylene monoimide dye-sensitised TiO_2_ in water†
†Electronic supplementary information (ESI) available: Experimental section, supporting tables and figures. See DOI: 10.1039/c8sc05693e


**DOI:** 10.1039/c8sc05693e

**Published:** 2018-12-21

**Authors:** Julien Warnan, Janina Willkomm, Yoann Farré, Yann Pellegrin, Mohammed Boujtita, Fabrice Odobel, Erwin Reisner

**Affiliations:** a Christian Doppler Laboratory for Sustainable SynGas Chemistry , Department of Chemistry , University of Cambridge , Lensfield Road , Cambridge CB2 1EW , UK . Email: reisner@ch.cam.ac.uk; b Université LUNAM , Université de Nantes , CNRS , Chimie et Interdisciplinarité: Synthèse, Analyse, Modélisation (CEISAM) , UMR 6230 , 2 rue de la Houssinière , 44322 Nantes cedex 3 , France . Email: fabrice.odobel@univ-nantes.fr ; Email: hamada.boujtita@univ-nantes.fr

## Abstract

Dye-sensitisation of TiO_2_ and other metal oxides is an established strategy to couple solar light harvesting with efficient charge separation for the production of electricity in dye-sensitised solar cells (DSCs) or fuels in dye-sensitised semiconductor photocatalysis (DSP). Perylene monoimide (PMI) dyes have emerged as promising organic dyes, but they have not previously been used in a functional assembly with TiO_2_ in aqueous solution. Here, five novel PMI dyes bearing carboxylic acid, phosphonic acid, acetylacetone, hydroxyquinoline or dipicolinic acid anchoring groups for attachment onto TiO_2_ are reported. We identified functional DSC and DSP systems with PMI-sensitised TiO_2_ in aqueous solution, which permitted a side-by-side comparison with respect to performance between the two systems. Structure–activity relationships allowed us to suggest anchor-condition-system associations to suit specific anchoring groups at various pH values, and with different electron mediators (redox couple or sacrificial electron donor) and catalysts in DSC and DSP schemes. A DSC sensitised with the hydroxyquinoline-modified PMI dye reached the highest short-circuit current density (*J*_SC_ ≈ 1.4 mA cm^–2^) in aqueous electrolyte solution during irradiation with simulated solar light. This dye also achieved a turnover number (TON_PMI_) of approximately 4900 for sacrificial proton reduction after 24 h irradiation in a DSP scheme with Pt as a H_2_-evolving co-catalyst at pH 4.5. This performance was only surpassed by the carboxylic acid-bearing dye, which reached a new benchmark turnover number (TON_PMI_ ≈ 1.1 × 10^4^ after 72 h) for an organic dye in nanoparticulate DSP for solar fuel production. At higher pH (8.5), our results showed that the phosphonic acid group allows for higher performance due to a stronger anchoring ability. This study provides a platform for aqueous PMI dye-sensitised TiO_2_ chemistry and gives valuable insights into the performance of different anchoring groups in DSC and DSP systems.

## Abstract 2

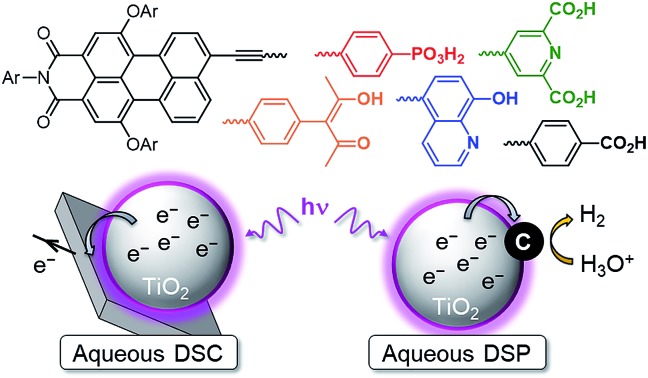
Anchor-bearing perylene monoimide dyes were synthesised and studied back-to-back in both aqueous dye-sensitised solar cells and semiconductor photocatalysis.

## Introduction

**Fig. 1:**
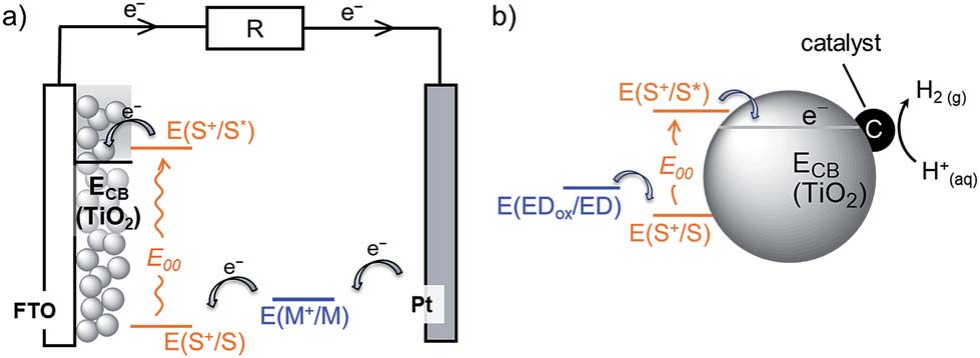
Schematic representation of solar energy conversion with dye-sensitised TiO_2_: (a) DSC^3^ and (b) DSP systems.^4^ See text for details.

Dye-sensitised semiconductor technology has emerged as a sustainable approach to produce renewable electricity in dye-sensitised solar cells (DSCs) and fuels in dye-sensitised photocatalysis (DSP) from abundant sunlight (Fig. 1).^1^–^6^ In a DSC, a photovoltage is produced through the photoexcitation of a sensitiser (S) anchored on a semiconductor electrode, followed by efficient charge injection into the semiconductor. These extracted charges are shuttled to a counter electrode (typically Pt), where they react with a diffusional redox mediator (M) that ultimately regenerates the ionised sensitiser. Analogously, solar irradiation of a DSP system results in accumulation of photoinjected electrons in the conduction band (CB) of the semiconducting particle. These electrons can subsequently be released to a co-immobilised catalyst to perform fuel synthesis (*e.g.* H_2_ production from proton reduction).^4^,^6^–^8^ The vast majority of DSP systems currently still rely on sacrificial electron donors (EDs) to regenerate the oxidised dye.^4^,^9^

Operation in water is desirable for dye-sensitised technologies as this potentially reduces costs, flammability, instability and environmental incompatibility.^10^ Despite seminal reports on aqueous DSCs in the 1980s, organic solvents (*e.g.* acetonitrile) have been predominantly used during the past decades as they enable high efficiencies *via* dye and mediator engineering.^3^,^11^–^14^ However, increasing attention to fully green processes has driven growing efforts towards aqueous DSCs, notably targeting optimised electrolytes (*e.g.* redox mediator and additives), improved surface wettability or water-tolerant dyes.^15^–^23^ DSP for water splitting is naturally inclined towards an aqueous solution as water can act as both solvent and substrate for H_2_ and O_2_ evolution.^4^

Nanoparticulate TiO_2_ is the prototype semiconductor as it is inexpensive, stable, displays excellent charge transfer kinetics and has suitable energy levels for DSCs and DSP.^24^ The dye is the second pivotal component in DSC and DSP technologies as it collects photons and governs the kinetics of charge injection into the semiconductor, as well as dye regeneration and charge recombination. Nevertheless, dyes have rarely been optimised for efficient performance in an aqueous dye-semiconductor environment, and therefore typically show low device efficiencies under such conditions.^10^,^17^ In this context, organic chromophores have notable advantages over Ru-based dyes in terms of abundancy of their atoms, tunability and strong π–π* transitions, making them suitable candidates for dye-sensitised technologies.^25^–^29^

**Chart 1:**
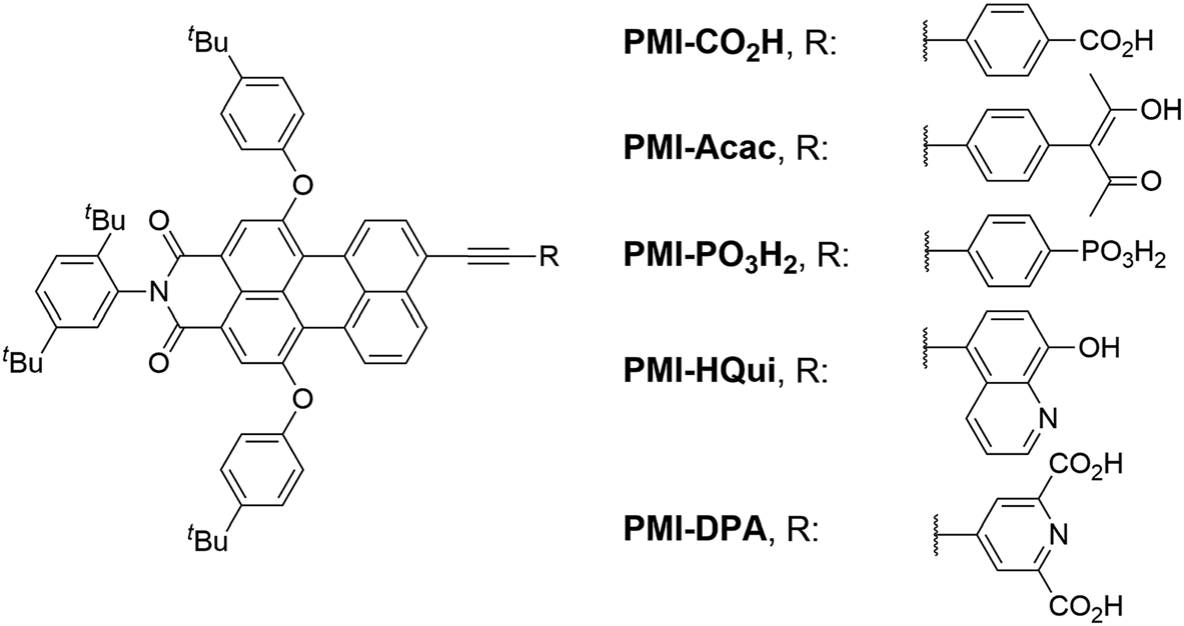
Chemical structure of PMI dyes with different anchoring groups used in this study.

Here, we report the synthesis of five novel perylene monoimide (PMI) dyes and their incorporation into aqueous DSCs and H_2_-producing nanoparticulate DSP systems. PMI chromophores benefit from an established synthetic protocol, adjustable electronic and photophysical properties, good light stability and high photovoltaic performance.^30^–^34^ The synthesised PMI dyes were characterised by ^1^H NMR, ^13^C NMR, ^31^P NMR spectroscopy and HRMS, and their optoelectronic properties were assessed using electrochemistry and UV-vis spectroscopy. The five PMI dyes differ in their anchor functionality for binding to TiO_2_, bearing either a carboxylic acid (**PMI-CO_2_H**), a phosphonic acid (**PMI-PO_3_H_2_**), an acetylacetone (**PMI-Acac**), a hydroxyquinoline (**PMI-HQui**) or a dipicolinic acid (**PMI-DPA**) group (Chart 1). The anchor moiety affects not only the electronic properties of the PMI, but also modulates proximity, binding strength and electronic communication at the dye-semiconductor interface. In addition, the backbone of the chosen PMI dyes contain bulky hydrophobic units to limit deleterious dye aggregation and to minimise desorption in aqueous media.^35^,^36^

Even though the nature of the anchor functionality is a vital part of the sensitiser in DSC and DSP, it has rarely been investigated in aqueous media, or using the same chromophore unit in DSC and DSP.^37^,^38^ The five PMI dyes were systematically studied side-by-side in DSC and DSP systems, at basic, neutral and acidic pH, in combination with I_3_^–^/I^–^, ascorbic acid (AA) or triethanolamine (TEOA) as mediators or sacrificial EDs. The diversity of studied systems, electron donors and pH values provides decisive information for the future design of dye-sensitised technology.

## Results and discussion

### Synthesis of PMI dyes

**Scheme 1:**
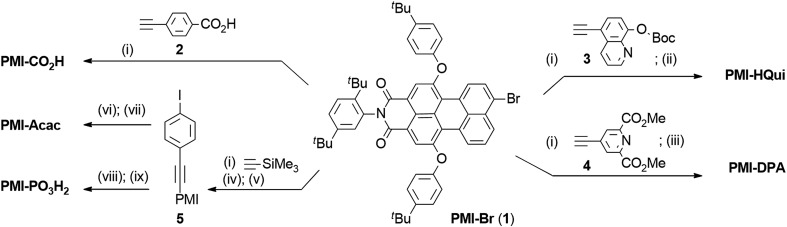
Synthesis of the PMI dyes with different anchoring groups. (i) Pd(PPh_3_)_4_, CuI, toluene, DIPEA or Et_3_N, [45 to 60] °C, [3 to 22] h; (ii) piperidine, DCM, r.t., 5 min; (iii) K_2_CO_3_, THF/H_2_O, 50 °C, 16 h; (iv) K_2_CO_3_, DCM/MeOH, r.t., 3 h; (v) 1,4-diiodobenzene, Pd(PPh_3_)_4_, CuI, toluene, Et_3_N, 70 °C, 2 h; (vi) DMIBE, Pd(PPh_3_)_4_, Cs_2_CO_3_, toluene/MeOH, 50 °C, 5 h; (vii) (a) Mo(CO)_6_, toluene/CH_3_CN/H_2_O, 90 °C, 3 h; (b) oxalic acid dihydrate, THF/H_2_O, 80 °C, 16 h; (viii) HPO_3_Et_2_, Et_3_N, Pd(PPh_3_)_4_, THF, 60 °C, 24 h; (ix) (a) Me_3_SiBr, Et_3_N, DCM, 50 °C, 5 h; (b) MeOH, r.t., 16 h. See ESI† for synthesis and characterisation of compounds **3** and **4**.

The preparation of the PMI dyes is based on a divergent modular synthetic approach starting from the brominated perylene monoimide **PMI-Br** (**1**) (Scheme 1).^35^ A Pd-catalysed Sonogashira cross-coupling reaction between **PMI-Br** and ethynyl derivatives bearing different anchoring groups generated the desired products. **PMI-CO_2_H** was obtained in 93% yield by coupling **PMI-Br** with 4-ethynylbenzoic acid (**2**). **PMI-HQui** and **PMI-DPA** were synthesised by reacting **PMI-Br** with 8-*tert*-butoxycarbonyloxy-5-ethynylquinoline (**3**) and 4-ethynyldipicolinic methyl ester (**4**), followed by cleavage of the Boc protecting group and ester hydrolysis in 46% and 33% overall yield, respectively.

The synthesis of **PMI-Acac** and **PMI-PO_3_H_2_** started from coupling of **PMI-Br** with ethynyltrimethylsilane followed by the cleavage of the trimethylsilyl group. A second Sonogashira reaction was subsequently performed between the resulting ethynyl-substituted PMI and an excess of 1,4-diiodobenzene to afford intermediate **5** in 81% yield. **PMI-Acac** was synthesised in two steps: a Suzuki–Miyaura reaction between **5** and 3,5-dimethylisoxazole-4-boronic acid pinacol ester (DMIBE) was followed by the opening of the isoxazole ring using [Mo(CO)_6_], and hydrolysis of the intermediate β-ketoenamine to give **PMI-Acac** (77% overall).^39^ The **PMI-PO_3_H_2_** sensitiser was also obtained in two steps *via* a palladium-catalysed Hirao reaction from the iodo-PMI derivative **5** and diethyl phosphite, followed by hydrolysis of the formed phosphonate ester using Me_3_Si–Br and MeOH affording **PMI-PO_3_H_2_** in an overall yield of 93%.

### Electronic properties

The influence of the different anchoring groups on the electronic properties of the PMI photosensitisers was studied by electronic absorption spectroscopy in *N*,*N*-dimethylformamide (DMF) solution. All PMI dyes displayed a broad and intense (*ε*_max_ > 3.5 × 10^4^ M^–1^ cm^–1^) absorption in the visible part (from 450 to 650 nm) of the solar spectrum (Fig. S1†) with an absorption maximum at 535 nm and a shoulder at 500 nm. No significant difference was observed between the dyes' visible absorption, which suggests that the anchors do not directly affect the PMI transition. An exception is **PMI-HQui**, which displays a red-shifted absorption maximum (*λ*_max_ = 545 nm) owing to the presence of a charge transfer band between the electron-rich quinoline unit and the PMI core. Consequently, this feature enables **PMI-HQui** to absorb photons from a wider range of the visible part of the solar spectrum.

**Fig. 2:**
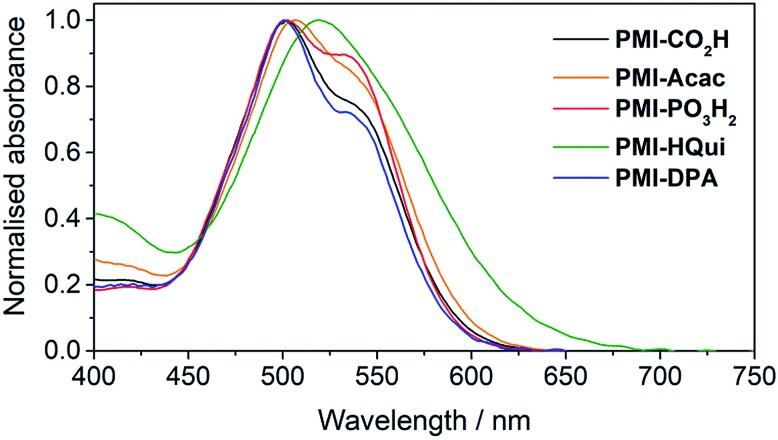
UV-Vis spectra of PMI dyes immobilised on a thin TiO_2_ film (6 μm thickness) recorded at room temperature.

In order to obtain a better assessment on the light harvesting ability of the DSC and DSP systems, we recorded the absorption spectra of the PMI dyes (Fig. 2) upon immobilisation on 6 μm-thick, mesoporous anatase TiO_2_ electrodes. The TiO_2_ electrodes were prepared from an anatase paste, following previously published procedures, and sensitised by soaking the electrodes in a 0.25 mM solution of the PMI dye in DMF overnight (see ESI† for details). On TiO_2_, the dyes maintained a broad absorption in the visible spectrum, whereas *λ*_max_ was generally observed at approximately 500 nm with a shoulder at 535 nm. Although also potentially affected by the protonation state of the anchoring group after immobilisation, this inversion behaviour of the two maxima intensities (at 500 and 535 nm) is in line with PMI aggregation affecting vibronic peak intensities.^40^,^41^

Electrochemical experiments were performed in a mixed dichloromethane (DCM)/DMF (95/5, v/v) solution – used instead of pure DMF conditions in order to reach higher dye concentration – with tetrabutylammonium hexafluorophosphate (TBAP, 0.1 M) as the supporting electrolyte. All cyclic voltammograms display an irreversible wave, located (onset potential) at approximately +1.4 V *vs.* normal hydrogen electrode (NHE) corresponding to the oxidation of the perylene core. This anodic wave is observed at more negative values in **PMI-HQui** (*E*_ox_(**PMI-HQui**) = +1.2 V *vs.* NHE), and at slightly more positive values in case of the **PMI-DPA** (*E*_ox_(**PMI-DPA**) ≈ +1.5 V *vs.* NHE). In the former case, this shift can be explained by the electron-rich hydroxyquinoline unit which destabilises the highest occupied molecular orbital energy level. The electron withdrawing effect of dipicolinic acid can explain the more difficult oxidation of **PMI-DPA**.

Fluorescence spectra of the PMI sensitisers were recorded in a diluted DMF solution, and the energy of the 0–0 transition values (*E*_00_) were estimated at approximately 2.22 eV by using the intersection between the normalised absorption and luminescence spectra (Fig. S2†). The different anchors do not strongly affect *E*_00_ values, giving oxidation potentials in the excited state (*E*(S^+^/S*)) between –1.01 and –0.75 V *vs.* NHE corresponding to **PMI-HQui** and **PMI-DPA**, respectively (Table 1).

**Table 1:** Maximum absorption wavelength (*λ*_max_), *E*_00_, the first oxidation potential (*E*(S^+^/S)) and (*E*(S^+^/S*)) of PMI dyes with different anchors

Dye	*λ*_max_ (nm)^*a*^/(*ε*, M^–1^ cm^–1^)	*E*_00_ (eV)	*E*(S^+^/S) (V *vs.* NHE)	*E*(S^+^/S*)^*b*^(V *vs.* NHE)
**PMI-CO_2_H**	536 (4.9 × 10^4^)	2.21	1.44	–0.77
**PMI-Acac**	538 (5.4 × 10^4^)	2.22	1.34	–0.88
**PMI-PO_3_H_2_**	536 (3.8 × 10^4^)	2.21	1.43	–0.78
**PMI-HQui**	545 (5.6 × 10^4^)	2.21	1.20	–1.01
**PMI-DPA**	536 (4.0 × 10^4^)	2.24	1.49	–0.75

^*a*^In DMF.

^*b*^ *E*(S^+^/S*) = *E*(S^+^/S) – *E*_00_. S: ground state of PMI, S*: excited state, S^+^: oxidised state.

The photoactivity mechanism of n-type DSC and DSP systems relies on an oxidative quenching of the sensitiser excited state (S*) by the conduction band of the semiconductor, followed by the regeneration of the dye ground state by the redox mediator or ED (Fig. 1).^4^ Considering the oxidation potentials of the redox mediator (*E*(I_3_^–^/I^–^) = 0.54 V *vs.* NHE) and sacrificial EDs (*E*(TEOA^+^/TEOA) = 0.82 V *vs.* NHE, pH 7.0; *E*(AA^+^/AA) < 0.20 V *vs.* NHE, pH 4.5) used in our study, dye regeneration is highly exergonic and therefore thermodynamically favourable.^42^,^43^ Similarly, a conduction band potential of TiO_2_, *E*_CB_(TiO_2_), of approximately –0.55 V *vs.* NHE (at pH 4.5) indicates a thermodynamically favourable electron injection.^44^ However, the driving force could become insufficient as the pH increases and *E*_CB_(TiO_2_) edge becomes more negative (shift of –59 mV per pH unit increase) (see Table S1†).^45^,^46^

### Dye-sensitised electrodes

We investigated and compared the photo-conversion efficiency of PMI-sensitised DSCs, in water and acetonitrile (ACN) as an organic solvent benchmark, using sensitised TiO_2_ anodes (100% anatase, 0.25 cm^2^, thickness 16 μm) in combination with I_3_^–^/I^–^ as the redox mediator and Pt as a counter electrode (Tables 2 and S2†). Digital photography of the sensitised electrodes and of an assembled DSC are available in Fig. S3.† Measurements were performed at room temperature without mask under simulated solar light (AM 1.5 G, 100 mW cm^–2^). Photovoltaic efficiencies (*η*s) from <0.1% to 0.5% were recorded in aqueous electrolyte solution, highlighting a substantial difference in performance between the PMI dyes with different anchors (Table 2). The short-circuit photocurrents (*J*_SC_s) are in line with previously reported Ru complex-sensitised aqueous DSC systems.^10^

**Table 2:** Photovoltaic performances of DSC devices dyed with **PMI-CO_2_H**, **PMI-PO_3_H_2_**, **PMI-Acac**, **PMI-HQui** or **PMI-DPA**^*a*^

Dye	*J*_SC_ (mA cm^–2^)	*V*_OC_ (mV)	FF (%)	*η* (%)
**PMI-CO_2_H**	0.85 ± 0.40	470 ± 30	69 ± 6	0.28 ± 0.30
**PMI-Acac**	0.24 ± 0.10	380 ± 8	60 ± 3	0.06 ± 0.01
**PMI-PO_3_H_2_**	0.70 ± 0.20	450 ± 30	35 ± 4	0.13 ± 0.10
**PMI-HQui**	1.37 ± 0.60	510 ± 5	68 ± 1	0.47 ± 0.30
**PMI-DPA**	1.30 ± 0.10	480 ± 10	69 ± 1	0.42 ± 0.10

^*a*^Conditions: aqueous electrolyte solution, redox mediator I_3_^–^/I^–^, 1 Sun, AM 1.5 G, after 9 d (see ESI for details).

The obtained *η*s are still low compared to top performing organic systems, or to the highest *η* value recorded (∼4%) with an iodine based aqueous electrolyte and the organic dye D149.^15^ This can be partially attributed to the electron-withdrawing ability of the PMI core that would limit electron injection and promote charge recombination. Nevertheless, this work constitutes the first example of a PMI-based aqueous DSC, and further optimised electrolytes and TiO_2_ surfaces previously reported for aqueous DSCs are likely to enable higher performances in the future.^10^,^46^,^47^

The main discrepancies between the performance of the dyes originate from variations in *J*_SC_, which follow the order **PMI-Acac** ≪ **PMI-PO_3_H_2_** ≈ **PMI-CO_2_H** < **PMI-DPA** ≤ **PMI-HQui**. Incident photon-to-current conversion efficiency (IPCE) measurements (Fig. S4†) also confirmed this order with **PMI-HQui** DSCs displaying the highest and broadest photon conversion efficiencies in line with the broader absorption of the dye (Fig. 2). As a result, the highest DSC efficiencies were obtained with **PMI-HQui** in both H_2_O and ACN conditions. The low *J*_SC_ and IPCE obtained with the **PMI-Acac**-sensitised DSCs, in both water- and ACN-based electrolyte solution, are primarily accounted for by inefficient electron injection. This can be explained by the limited orbital overlap due to the perpendicular orientation of the anchor with respect to the phenyl plane, which impedes any electron-withdrawing effect from the anchor and ultimately reduces electron density close to the TiO_2_ surface.^48^,^49^

The similar *J*_SC_ and open-circuit voltage (*V*_OC_) values obtained in water for the dyes bearing carboxylic, phosphonic and dipicolinic acids reflects their comparable photosensitising abilities. However, the low fill factors (FFs) recorded on **PMI-PO_3_H_2_**-based DSCs infer substantial charge recombination at the semiconductor–electrolyte interface. Interestingly, upon replacing H_2_O for ACN this photosensitiser produces high *J*_SC_s and FFs similar to the other PMI-dyes, revealing a specific deleterious impact/interaction of H_2_O with the phosphonic acid and the surface of TiO_2_ (Table S2†), leading to high recombination phenomena and series resistance.^36^

In order to obtain further insights on the potential of the anchors in water, we evaluated the performance of the dyes on the TiO_2_ photoanodes under an applied potential (in a three-electrode setup). Two commonly employed electron donors, AA and TEOA, were used instead of the DSC redox mediator I_3_^–^/I^–^.^43^ These experiments, ‘half way’ between DSC and DSP systems, allow for a preliminary evaluation of the sensitisers' capability to extract charge from EDs in a DSP system through application of a positive applied bias (*E*_app_), which limits geminate and non-geminate recombination.

The experiments were conducted using a dye-sensitised TiO_2_ film coated on a fluorine tin oxide-coated glass slide as a working electrode, a Hg/HgSO_4_ reference electrode (*E*^0^(*vs.* NHE) = *E*^0^(*vs.* Hg/HgSO_4_) + 0.64 V) and a platinum wire counter electrode. Linear sweep voltammetry conducted under chopped illumination (2 W m^–2^) showed constant *J* between 0.64 to 0.14 V *vs.* NHE for all dyes (Table 3, Fig. S5 and S6†). Photocurrents between 0.2 and 0.8 mA cm^–2^, displaying a similar trend to those obtained in the DSC configuration, were observed in aqueous AA solution (pH 4.5, Tables 2 and 3). This demonstrates the ability of AA to act as an ED for all sensitisers, although at a somewhat reduced performance compared to I_3_^–^/I^–^. Photocurrents attained with **PMI-PO_3_H_2_**-sensitised electrodes are significantly lower than those observed in a DSC configuration, which alludes to complications in the charge extraction process, in agreement with the low FF recorded in aqueous DSC (Table 2).

**Table 3:** Biased photoelectrochemical performance of TiO_2_ electrodes sensitised with the PMI dyes in presence of an ED and recorded under a 2 W m^–2^ white light^*a*^

Dye	*J* (mA cm^–2^)	
	AA (pH 4.5) *E*_app_ = 0.24 V *vs.* NHE	TEOA (pH 8.5) *E*_app_ = 0.64 V *vs.* NHE
**PMI-CO_2_H**	0.38	0.025
**PMI-Acac**	0.24	0.009
**PMI-PO_3_H_2_**	0.26	0.024
**PMI-HQui**	0.80	0.017
**PMI-DPA**	0.50	0.018

^*a*^General conditions: aqueous electrolyte, ED = AA or TEOA, 2 W m^–2^ white light irradiation (see ESI for details).

Substantially lower *J*s (≤0.025 mA cm^–2^) were recorded in the presence of TEOA as ED at pH 8.5 (Fig. S6†), in comparison to those obtained in AA-containing solutions at pH 4.5. This could be first explained by the lack of exergonicity in the electron injection process as illustrated by the small Gibbs free energy values (Table S1†) in basic conditions. Nevertheless, the order of *J* is not directly reflected by the Gibbs energy within the different dyes which could allude towards additional limiting factors. Interestingly, the *J*s of the cells level out at ∼0.020 mA cm^–2^ with very little discrepancies between the dyes (except **PMI-Acac**), which suggests the existence of a major kinetically limiting step, ascribed to slow dye regeneration by TEOA. As a result, the accurate evaluation of the impact of the anchors on performance is difficult under such conditions; however, it is clear that the acetylacetone anchor delivers lower photocurrents suggesting even further restrictions in the charge injection.

In order to study the differences in dye regeneration kinetics from AA and TEOA, we performed two additional measurements using **PMI-CO_2_H** as a model dye using AA as ED in neutral and basic conditions at *E*_app_ = 0.54 V *vs.* NHE. Similar *J*s of 0.48 and 0.47 mA cm^–2^ were obtained at 7.0 and 8.5, respectively, which is close to the one recorded at pH 4.5, *i.e.* 0.6 mA cm^–2^. This contrast with the much lower value recorded with TEOA at pH 8.5 (*J* = 0.02 mA cm^–2^ at *E*_app_ = 0.54 V *vs.* NHE) and suggest that the low regenerating ability of TEOA is a major limiting factor in our system rather than issues in electron injection.

Electrochemical impedance spectroscopy (EIS) measurements were performed on similar electrodes at open-circuit potential under white light illumination using AA and TEOA at pH 8.5. For both AA- and TEOA-based electrolytes, Bode plot traces revealed phase values close to zero for frequencies around 100 kHz. This indicates an ohmic resistive behaviour (Fig. S7a†), resulting from the sum of the electrolyte solution and defect of the photoelectrode resistances. In the middle frequencies region, phase values less than 90° were observed and attributed to charge transfer resistances (*R*_CT_) and a double layer capacitance at the **PMI-CO_2_H** dye/electrolyte interface. *R*_CT_ were deduced from Nyquist plots (Fig. S7b†) at around 1 and ≫20 kΩ for AA and TEOA solutions, respectively. These results indicate a better regeneration of S^+^ by AA than by TEOA with the latter appearing responsible for the lower photocurrents recorded at pH 8.5.

We subsequently also performed applied bias incident photon-to-current efficiency (ABCE) experiments under optimal conditions using AA (pH 4.5) as ED, at *E*_app_ = 0.24 V *vs.* NHE, in order to obtain insights on the wavelength-dependent efficiency of electron injection (Fig. S8†). The results confirmed that the cells sensitised with **PMI-HQui** and **PMI-Acac**/**PMI-PO_3_H_2_** displayed the highest and lowest photon-to-electron efficiencies of 30 and 6–7% respectively at their respective maximum absorption. Compared to the other dyes, the former showed a broader light sensitivity over the solar spectrum, and demonstrated the highest conversion of photons. This observation is in line with the higher *J*_SC_s recorded in DSC configuration.

### Assembly of DSP system

Pt was used as a H_2_-evolving catalyst in order to assess the dye performances in absence of catalyst-associated limitations, and was pre-deposited on TiO_2_ nanoparticles (P25, Evonik Industries) as previously described.^37^ The PMI-modified TiO_2_|Pt nanoparticles (PMI|TiO_2_|Pt) were assembled by sonicating the pre-platinised-TiO_2_ particles in a dilute solution of PMI dye in DMF (see ESI† for details). The PMI|TiO_2_|Pt particles were separated from solution after one hour *via* centrifugation, and the supernatant was analysed by UV-Vis spectroscopy to quantify the amount of immobilised dye on TiO_2_ (Table S3† and Fig. S9†). Approximately 85% of **PMI-Acac**, **PMI-DPA** and **PMI-HQui** (≈33 nmol) available in the original dyeing solution (*n*_tot_ ≈ 39 nmol) were attached onto 2 mg of TiO_2_|Pt. This result indicates a strong binding ability of these anchors with a modest influence of the steric hindrance or footprint of the anchor. The loading increases and decreases in the case of **PMI-PO_3_H_2_** (>95%) and **PMI-CO_2_H** (≈65%), respectively, demonstrating the superior anchoring ability of the phosphonic acid. The immobilisation of the PMI dyes was also confirmed by ATR-FTIR spectroscopy of the sensitised nanoparticles with the spectra revealing clear *ν*(C=O)_amide_ and *ν*(C=C)_aromatic_ bands of the PMI-dyes at 1710 and 1650 cm^–1^, respectively (Fig. S9†).

### Photocatalytic activity

Photocatalytic experiments were carried out in water at pH 4.5, 7.0 and 8.5 using either AA (pH 4.5, 0.1 M), or TEOA (pH 7.0 and 8.5, 0.1 M) as both buffer and ED.^50^–^53^ For photocatalytic experiments, the PMI|TiO_2_|Pt nanoparticles were dispersed in the aqueous ED solution (1.25 mg of pre-modified particles in 3 mL of ED solution) in a sealed photoreactor *via* sonication for 15 min, then purged with N_2_ (including 2% CH_4_ as internal gas chromatography standard), and irradiated with UV-filtered simulated solar light (AM 1.5 G, 100 mW cm^–2^, *λ* > 420 nm). Light-driven H_2_ evolution was monitored in regular time intervals by gas chromatography. Control experiments in absence of dye revealed no H_2_ or only negligible amounts of H_2_ are evolved.^28^

The initial dye-based turnover frequencies (TOF_PMI_ after 1 h) in aqueous DSP with PMI|TiO_2_|Pt in pH 4.5 AA (0.1 M) for solar H_2_-evolution agree well with trends observed in the aqueous DSCs (see above): **PMI-Acac** < **PMI-PO_3_H_2_** < **PMI-DPA** ≈ **PMI-CO_2_H** < **PMI-HQui** (Table 4 and S4, and Fig. S10†). **PMI-Acac** shows the lowest performance, which is most likely due to the unfavourable orientation of the Acac anchoring group. **PMI-HQui** displays the highest initial TOF_PMI_, but it also exhibits the largest drop in photoactivity over time followed by **PMI-DPA** (Table S5†). The activity of the other PMI|TiO_2_|Pt systems remained generally constant during 7 h of irradiation (Fig. S10†). This indicates degradation of the **PMI-HQui** and **PMI-DPA** dye/anchor, or loss of dye molecules from the surface leading to a decrease in performance over time.

**Table 4:** Photocatalytic performance of PMI|TiO_2_|Pt

System^*a*^	TOF_PMI_/h^–1^ (1 h)^*b*^	*n*(H_2_)/μmol (24 h)	TON_PMI_ (24 h)^*b*^
**pH 4.5**			
**PMI-CO_2_H**	344 ± 38	53.7 ± 6.2	6461 ± 749
**PMI-Acac**	112 ± 12	21.7 ± 2.2	2146 ± 203
**PMI-PO_3_H_2_**	210 ± 27	42.5 ± 6.3	3546 ± 523
**PMI-HQui**	467 ± 72	53.3 ± 5.9	4928 ± 549
**PMI-DPA**	305 ± 59	41.4 ± 2.9	3943 ± 394

**pH 7**			
**PMI-CO_2_H**	59.2 ± 5.9	3.9 ± 0.5	471 ± 63
**PMI-Acac**	10.9 ± 1.0	1.3 ± 0.1	133 ± 13
**PMI-PO_3_H_2_**	27.4 ± 2.7	3.6 ± 0.4	303 ± 30
**PMI-HQui**	25.6 ± 2.6	2.5 ± 0.5	232 ± 26
**PMI-DPA**	27.5 ± 2.7	3.8 ± 0.2	366 ± 37

**pH 8.5**			
**PMI-CO_2_H**	58.6 ± 14.5	4.1 ± 1.4	490 ± 170
**PMI-Acac**	23.4 ± 3.1	3.0 ± 0.7	294 ± 67
**PMI-PO_3_H_2_**	60.9 ± 6.1	8.5 ± 1.3	708 ± 107
**PMI-HQui**	26.4 ± 2.8	2.8 ± 0.4	262 ± 36
**PMI-DPA**	32.8 ± 3.3	4.7 ± 0.7	444 ± 62

^*a*^Conditions: 1.25 mg PMI|TiO_2_|Pt in 3 mL ED solution (0.1 M of AA or TEOA), UV-filtered simulated solar irradiation (AM 1.5 G, 100 mW cm^–2^, *λ* > 420 nm, 25 °C).

^*b*^TOF_PMI_ (1 h) and TON_PMI_ were calculated based on the loading of the TiO_2_|Pt nanoparticles (see Table S3).

**Fig. 3:**
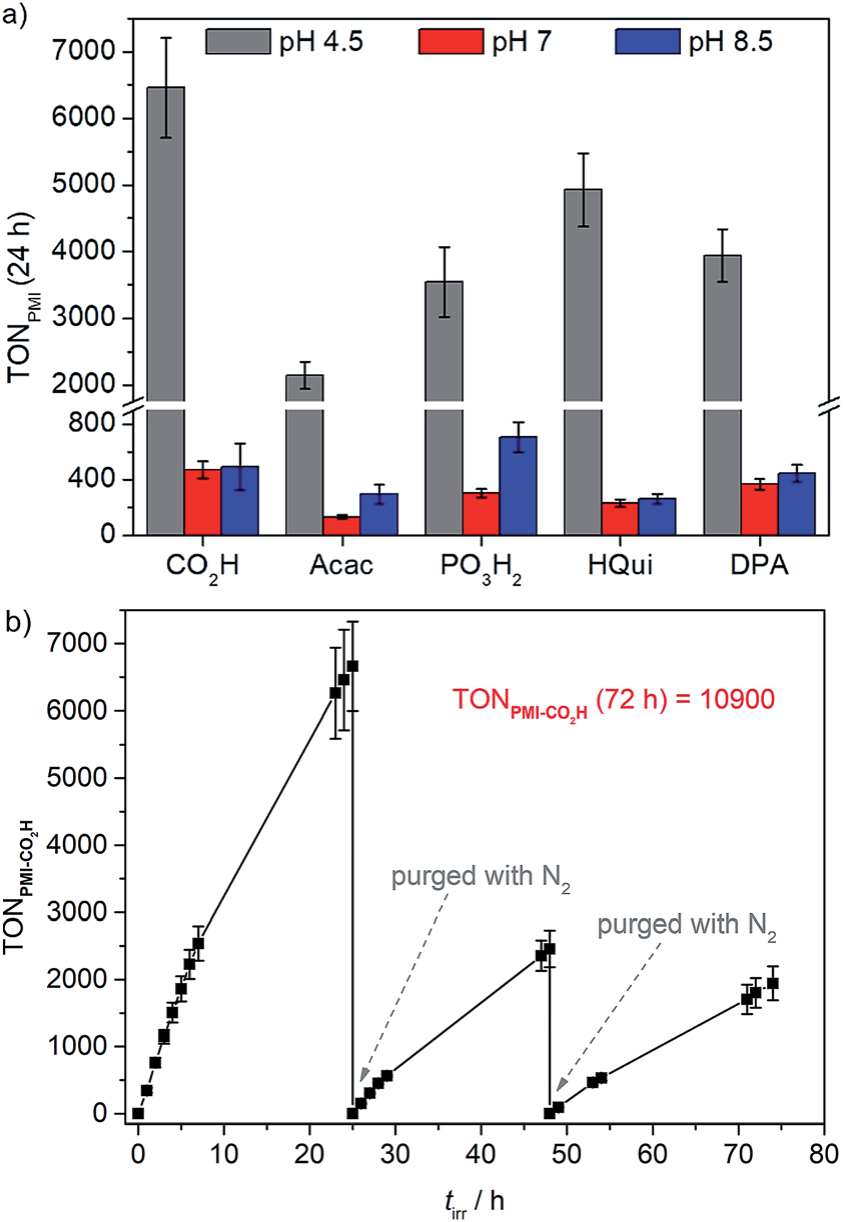
(a) Photocatalytic activity of PMI|TiO_2_|Pt expressed as TON_PMI_ after 24 h of irradiation in pH 4.5 AA (grey), pH 7 TEOA (red), and pH 8.5 TEOA (blue) solution (0.1 M each); (b) long-term experiment (TON**_PMI-CO2H_***vs. t*_irr_) using **PMI-CO_2_H** in pH 4.5 AA solution. Samples were purged with N_2_ after 24 and 48 h. Conditions: UV-filtered simulated solar light irradiation (AM 1.5 G, 100 mW cm^–2^, *λ* > 420 nm) of 1.25 mg PMI|TiO_2_|Pt in 3 mL ED (AA or TEOA) solution.

Up to 54 μmol of H_2_ were produced with PMI|TiO_2_|Pt, with **PMI-CO_2_H** giving an initial TOF of 344 h^–1^ and the highest TON_PMI_ of approximately 6460 after 24 h of UV-filtered simulated solar light irradiation (Table 4, Fig. 3a). This is significantly higher than the TON obtained for our recently reported phosphonated diketopyrrolopyrrole (DPP) or Ru(2,2′-bipyridine)-based dye-sensitised TiO_2_|Pt systems, where a maximum TON_DPP_ of 2660 and initial TOF_DPP_ of 337 h^–1^ were obtained under the same experimental conditions.^28^ The similar initial TOFs of the two systems and the higher final TON_PMI_ could reflect on superior stability of the PMI core compared to the DPP. A long-term experiment was performed with **PMI-CO_2_H** showing that the PMI|TiO_2_|Pt system, despite experiencing some dye degradation, remained active for over 72 h of light irradiation. A cumulative TON_PMI_ of approximately 1.1 × 10^4^ (Fig. 3b) was achieved, which corresponds to the highest TON obtained for an organic dye in an aqueous nanoparticulate DSP system.

The compatibility of PMI dyes with a molecular catalyst was studied with a phosphonated DuBois-type nickel bis(diphosphine) complex (**NiP**, Fig. S11†).^51^**NiP** was added to Pt-free, **PMI-CO_2_H**-sensitised TiO_2_ nanoparticles, which were suspended in aqueous AA solution prior to addition. The deaerated **PMI-CO_2_H**|TiO_2_|**NiP** assembly achieved a TON**_NiP_** and TON_PMI_ of approximately 110 and 170 after 24 h of irradiation, respectively (Fig. S12†). Although slightly lower, this performance for catalytic H_2_ production agrees well with previously reported TONs for DPP-based DSP systems using **NiP** as catalyst (TON_DPP_ = 204 (21 h) *vs.* TON_PMI_ = 170 (24 h)),^28^,^51^ and illustrates that Pt can be replaced to give a precious metal-free PMI|TiO_2_|catalyst system. The lower activity reached with the molecular catalyst compared to Pt was previously attributed to the slower kinetics of the former, that favours charge recombination from the e^–^ in the TiO_2_ CB to the oxidised dye.^28^ When **NiP** is employed, the lower activity of the PMI-dyes compared to the DPP-dyes alludes towards faster charge recombination in the former case that can be attributed to the dye design as discussed above.

The photocatalytic performance of PMI|TiO_2_|Pt was subsequently studied with TEOA (pH 7 and 8.5) as an ED. The activity is significantly lower compared to the AA-containing systems (Table 4, S6 and S7,† and Fig. 3a, S13 and S14†). Although the order of efficiency does not clearly reflect the Gibbs energies (Table S1†), these results match the trend obtained from photocurrent measurements of PMI-sensitised TiO_2_ photoanodes and is probably due to more difficult dye regeneration by the ED and a more difficult electron injection at higher pH. The generally lower performance recorded at pH 7.0 compared to pH 8.5 has been ascribed to the coexistence of protonated and neutral forms of TEOA due to its p*K*_a_ value within the pH range investigated (p*K*_a_ = 7.9). As the pH of the solution decreases the equilibrium favours the existence of the protonated form of TEOA which is an inferior donor and therefore impedes the regeneration of the oxidised sensitiser.^54^

PMI dyes with acid-containing anchoring groups appear beneficial under these more basic conditions with **PMI-CO_2_H** and **PMI-PO_3_H_2_** achieving a TON_PMI_ of approximately 470 and 710 at pH 7.0 and pH 8.5, respectively. Specifically, the phosphonic acid anchoring group has been previously reported as a more stable anchor at higher pH values in comparison to carboxylic acids.^4^,^37^ This advantage is illustrated by the strong photoactivity drop during the first 7 h for **PMI-CO_2_H** (≈37%, Fig. S13 and S14†), whereas the other anchoring groups (Acac, DPA, PO_3_H_2_ (≈15–25% loss)) appear more robust under the pH neutral/alkaline conditions. The almost pH-independent stabilities (Table S5 and Fig. S15†) observed for the **PMI-HQui** and **PMI-DPA** dyes likely originate from dye degradation or from the system progressive aggregation (observed after experiments), rather than desorption or limitations from the regeneration of S^+^ by the EDs. The low activity observed for the **PMI-HQui** (TON**_PMI-HQui_** = 232 and 262 at pH 7.0 and 8.5, respectively) and generally fast deactivations highlight issues arising from fast desorption/degradation.

## Conclusions

We report the synthesis of five novel PMI photosensitisers with different anchoring groups, and their successful integration into solar light-driven systems towards electricity and H_2_ production in pure aqueous solution. Varying the surface anchor group enabled a unique side-by-side comparison and highlights their advantages and weaknesses in aqueous DSC and DSP schemes. Despite high activities in acidic media, the hydroxyquinoline anchor is the most sensitive towards desorption/degradation under the chosen experimental conditions. Similarly, the carboxylic acid anchor delivers the highest photoactivity, and is a robust anchor in acidic and pH neutral media, but undergoes the fastest hydrolysis from the metal oxide surface in alkaline solution. Despite the hydrophobicity of the PMI dyes, a stronger anchor such as the phosphonic acid is required to allow for stable performance at alkaline pH.

We show that the same dye design rules apply to aqueous DSC and DSP technologies, with the individual system performance being similarly affected by the anchor of the dye and by the variation of the pH value of the solution. Consequently, we achieved promising photocurrents and good fill factors in aqueous DSC, as well as record TONs and impressive stability towards H_2_ evolution in DSP with a molecular dye-sensitised TiO_2_. This first generation of PMI dyes reveals significant insights drawn from structure–activity relationships, which will be applicable to a broad range of dye-sensitised technology in aqueous media. As this study focused specifically on the nature of the anchor and the dye–TiO_2_ interface, this work leaves promising opportunities for further dye improvements towards high performance under aqueous media such as implementing directional push–pull architectures, extending π conjugation or increasing the wettability of the TiO_2_ surface.

## Conflicts of interest

There are no conflicts to declare.

## Supplementary Material

Supplementary information
