# Supplementary material for: Solar electricity and fuel production with perylene monoimide dye-sensitised TiO2 in water
Source: Chem Sci. 2018 Dec 21;10(9):2758–66. doi: 10.1039/c8sc05693e (PMC6419928; doi:10.1039/c8sc05693e)
Supplement: Supplementary file 1 [file SC-010-C8SC05693E-s001.pdf]

Electronic Supplementary Material (ESI) for Chemical Science.
This journal is © The Royal Society of Chemistry 2019

Electronic Supporting Information

Solar Electricity and Fuel Production with Perylene Monoimide Dye-Sensitised TiO_2 in Water

Julien Warnan,[a] Janina Willkomm,[a] Yoann Farré,[b] Yann Pellegrin,[b] Mohammed Boujtita,[b]* Fabrice Odobel[b]* and Erwin Reisner[a]*

[a]Christian Doppler Laboratory for Sustainable SynGas Chemistry, Department of Chemistry, University of Cambridge, Lensfield Road, Cambridge, CB2 1EW, UK.

[b]Université LUNAM, Université de Nantes, CNRS, Chimie et Interdisciplinarité: Synthèse, Analyse, Modélisation (CEISAM), UMR 6230,2 rue de la Houssinière, 44322 Nantes cedex 3, France

*E-mail:

reisner@ch.cam.ac.uk (Web: http://www-reisner.ch.cam.ac.uk)
fabrice.odobel@univ-nantes.fr
hamada.boujtita@univ-nantes.fr

**Experimental Section**	**S1 – S17**
**Supporting Tables**	**S18 – S20**
**Supporting Figures**	**S21 – S27**
**Supporting References**	**S27**

Experimental Section

Materials and methods

Chemicals for analytical measurements were of the highest available purity. Aeroxide P25 TiO_2 particles (anatase/rutile: 8/2 mixture, average particle size = 21 nm) were provided by Evonik Industries. The TiO_2 paste (Dyesol DSL 18NR-T and Dyesol DSL 18NR-AO) was purchased from Dyesol. FTO sheets were purchased from Pilkington (TEC7, 6-8 Ω sq^{-1}). Platinised TiO_2 (TiO_2|Pt), $[Ni(P^{Ph}{}_2N^{PhCH_2PO(OH)_2}{}_2)_2]Br_2\cdot HBr$ (**NiP**), compounds **PMI-Br** (**1**), **3** and **10** were synthesised according to reported procedures.[1–5] Chemicals were purchased from Sigma-Aldrich or Alfa Aesar, and used as received. Thin-layer chromatography (TLC) was performed on aluminium sheets pre-coated with Merck 5735 Kieselgel $60F_{254}$. Column chromatography was carried out either with Merck 5735 Kieselgel 60F (0.040-0.063 mm mesh).

Physical characterisation

^1H, ^{13}C and ^{31}P NMR spectra were recorded on an *AVANCE 300 UltraShield BRUKER* and *AVANCE 400 BRUKER.* Chemical shifts for ^1H, ^{13}C NMR spectra are referenced relative to residual protium in the deuterated solvent ($CDCl_3$ δ = 7.26 ppm for ^1H and δ = 77.16 ppm for ^{13}C; THF-d^8 δ = 3.57, 1.72 ppm for ^1H and δ = 67.21, 25.31 ppm for ^{13}C; CD_3OD δ = 3.31 ppm for ^1H and δ = 49.00 ppm for ^{13}C), or to an internal reference (TMS, δ = 0 ppm for both ^1H and ^{13}C).[6] High-resolution mass (HR-MS) spectra were obtained either by electrospray ionization coupled with high resolution ion trap orbitrap (LTQ-Orbitrap, ThermoFisher Scientific) or by MALDI-TOF-TOF (Autoflex III, Bruker), working in ion-positive or ion-negative mode. UV-visible absorption spectra were recorded on a Variant Cary 300, using 1 cm path length cells. Emission spectra were recorded on a Fluoromax-4 Horiba Jobin Yvon spectrofluorimeter (1 cm quartz cells). The emission spectra were recorded with an absorbance of *circa* 0.05 on the most intense visible absorption band ([dye] ≈ 1 μM). Attenuated total reflectance FT-IR spectra of the compounds were recorded on a Nicolet iS50 spectrometer.

Electrochemical measurements

Electrochemical measurements were made under an argon atmosphere in the mixture CH_2Cl_2 (DCM)/DMF, 95/5 containing Bu_4NPF_6 (0.1 M). Cyclic voltammetry experiments were performed by using a SP300 Bio-Logic potentiostat/galvanostat. A standard three-electrode electrochemical cell was used. Potentials were referred to a saturated calomel electrode as internal reference and converted to NHE by adding +0.24 V.[7] The working electrode was a glassy carbon disk and the auxiliary electrode was a Pt wire. The scan rate was 100 mV s^{-1} in all experiments.

Chopped light photocurrent measurements were performed with an integrated photoelectrochemical workstation (ZAHNER Elektrik GmbH & Co. KG, Germany), containing a potentiostat for the light control and a second potentiostat (Zennium) for the electrochemical control all run under Thales software. A standard three electrode configuration with Hg/$HgSO_4$, coiled Pt-wire and a PMI-sensitised TiO_2 film (area 0.25 cm^2) deposited on FTO substrate (see preparation method below) were used as reference, counter and working electrodes, respectively. A white light lamp (1088wlr02, ZAHNER Elektrik GmbH & Co. KG, Germany) was used as the light source (2 W m^{-2}) and was periodically switched on and off, while the potential was swept at a rate of 5 mV s^{-1}.

Preparation of ED solutions

Triethanolamine (TEOA) hydrochloride was used to prepare the TEOA buffer solution (0.1 M, pH 7.0 or pH 8.5). Ascorbic acid (AA) solutions (0.1 M, pH 4.5) were freshly prepared for each experiment from L-ascorbic acid. The pH of each solution was adjusted with an aqueous NaOH solution. The final pH value was confirmed after diluting the ED solutions to the final concentration of 0.1 M.

Electrochemical Impedance Spectroscopy (ESI) Measurements

ESI was performed using an integrated photoelectrochemical workstation from Zahner (ZAHNER- Elektrik GmbH & Co. KG, Germany), containing a potentiostat for the light control and a second potentiostat (Zennium) for the electrochemical control. A standard three-electrode configuration using Hg/$HgSO_4$, a coiled Pt-wire and a PMI-sensitised TiO_2 film (area 0.25 cm^2) deposited on FTO substrate (see preparation method below) were used as reference, counter and working electrodes, respectively. Impedance plots were obtained by scanning frequencies between 100 kHz and 100 mHz using a 10 mV alternating current (AC) under open-circuit potential under white light illumination (lamp 1088wlr02, ZAHNER-Elektrik GmbH & Co. KG, Germany - (2 W m^{-2}).

Preparation and Testing of Dye-sensitised Solar Cells

FTO conductive glass substrates (F-doped SnO_2) were washed by successive sonication in soapy water, then an ethanolic solution of HCl (0.1 M) for 10 min, and finally dried in air. TiO_2 films were prepared in three steps. A first treatment is applied by immersion for 30 min in an aqueous $TiCl_4$ solution at 80 °C. Three successive layers of mesoporous TiO_2 were then screen printed using a transparent colloidal paste (Dyesol DSL 18NR-T) and a final light scattering layer (Dyesol DSL 18NR-AO) was affixed, with 20 min long drying steps at 150 °C between each layer. The obtained substrates were then sintered at 450 °C, following a progressive heating ramp (325 °C for 5 min, 375 °C for 5 min, 450 °C for 30 min). A second $TiCl_4$ treatment was immediately conducted afterwards and the electrodes were fired one

last time at 450 °C for 30 min. Thicknesses (16 µm) were measured by a Sloan Dektak 3 profilometer. The prepared TiO_2 electrodes were soaked while still hot (ca. 80 °C) in a 0.25 mM solution of the PMI dye in DMF (0.2 mM). After one night of dyeing, the electrodes were rinsed with DCM and dried in air, in the dark. Platinum based counter electrodes were prepared by drop casting two drops of hexachloroplatinic acid in distilled isopropanol (2 mg per mL) on FTO plates, and subsequent firing at 380 °C for 30 minutes. The photoelectrode and the counter electrode were placed on top of each other and sealed using a thin transparent film of Surlyn polymer (DuPont, 60 µm) as a spacer. The resulting chamber was filled with an iodine-based electrolyte. Acetonitrile electrolyte solutions contained 50 mM I_2, 0.1 M LiI and 0.6 M 1-ethyl-2,3-dimethylimidazolium iodide whereas aqueous electrolyte solutions contained 20 mM I_2, 4 M KI and saturated with chenodeoxycholic acid (CDCA) in water. The electrolyte was introduced in the cells by vacuum back filling through a predrilled hole in the counter electrode, and the photovoltaic device were sealed afterwards with Surlyn and a cover glass. The DSCs have an active area of 0.25 cm^2 and were tested without mask. Photovoltaic measurements were performed with a calibrated AM 1.5 artificial solar light simulator (Oriel) and a Keithley 2400 digital source-meter; data were collected with a local software designed by Synervia (labview). Before the measurements, the Oriel Lamp was calibrated to 100 mW cm^{-2} with a certified silicon cell covered with KG5 filter. All experiments were performed in quadruplicate (*i.e.* on four different DSCs) and the mean values and standard deviations (error) are reported.

Determination of Applied Bias Incident Photon to Current Efficiency (ABCE) and 3-electrode Chopped Light Measurements.

Measurements were recorded with a Zahner system (Zahner CIMPS) equipped with the tunable light source TLS03. A standard three electrode configuration with $Hg/HgSO_4$, coiled Pt-wire and a PMI-sensitised TiO_2 film (area 0.25 cm^2) deposited on FTO substrate (see preparation method above) were used as reference, counter and working electrodes respectively. During measurements, the TiO_2 films were exposed to white light illumination (lamp 1088wlr02, ZAHNER- Elektrik GmbH & Co. KG, Germany - (2 W m^{-2}) with a modulation frequency of 1 Hz. The electrolyte was either TEOA or AA buffer solutions containing 0.1 M $LiClO_4$.

Sensitisation of TiO_2 nanoparticles with PMI dyes

TiO_2 was platinised according to a published procedure.[1] Briefly, P25 TiO_2 particles (Evonik Industries) were dispersed in water (1 M methanol) and purged with N_2 for 30 min. Chloroplatinic acid (0.1 mM) was added and the suspension was irradiated with UV light (6 W) for 1 h. Particles were then separated *via* centrifugation, washed three-times with water and dried under high vacuum. Pre-platinised TiO_2 nanoparticles were dispersed in dry DMF

(0.85 mg mL^{-1}). The PMI dye was added (20 nmol (mg TiO_2|Pt)$^{-1}$, 1 mM in dry DMF). The resulting suspension was sonicated for 1 h at r.t. Particles were separated *via* centrifugation, and the supernatant analysed *via* UV-vis spectroscopy to determine the loading of the PMI onto the TiO_2|Pt nanoparticles. The sensitised particles were washed with dry DMF (3 x 5 mL), and then dried under vacuum overnight. The supernatant after the consecutive washing steps did not contain any desorbed PMI dye as determined by UV-vis spectroscopy. For experiments with the molecular catalyst **NiP**, unmodified TiO_2 was sensitised with PMI following the same procedure.

Photocatalytic experiments

PMI|TiO_2|Pt particles (1.25 mg) were dispersed in the ED-containing buffered solution (3 mL) *via* sonication for 15 min in a sealed photoreactor. Samples were kept in the dark, and purged with N_2 (2% CH_4 as internal standard for gas analysis) for 15 min. In the case of the molecular catalyst **NiP**, PMI|TiO_2 (1.25 mg) were dispersed in AA buffer solution (pH 4.5, 0.1 mM, 2.987 mL) *via* sonication for 15 min. **NiP** (12.5 nmol, 1 mM in methanol) was subsequently added, and the photoreactor then sealed and purged as described above. Samples were then irradiated with a calibrated LOT solar light simulator equipped with an UV- and IR water filter (AM 1.5G, 100 mW cm^{-1}, λ > 420 nm; irradiation area of DSP solution of approximately 3.3 cm^2). The samples were kept at 25 °C with a temperature-controlled water bath and stirred during the course of the reaction. The headspace of the photoreactor (4.84 mL) was sampled in regular time intervals and analysed *via* gas chromatography (GC). The gas chromatograph (Agilent 7890A Series) was equipped with a 5 Å molecular sieve column (held at 45 °C) and a thermal conductivity detector. Nitrogen was used as carrier gas at a flow rate of approximately 3 mL min^{-1}. The GC was calibrated in regular intervals to determine the response factor of H_2 against the internal CH_4 standard. All experiments were performed at least in triplicate (unless otherwise noted) and the mean values and standard deviations (error) are reported (see below for statistical analysis). A minimum of 10% error was assumed for all experiments.

$$\text{TON}_{\text{dye}} = \frac{n(\text{H}_2)}{n(\text{dye})} \times 2 \;\; ; \;\; \text{TON}_{\textbf{NiP}} = \frac{n(H_2)}{n(\textbf{NiP})}$$

where TON = turnover number, n(H_2) = moles of photo-generated H_2, n(dye) = moles of dye.

$$\text{TOF}_{\text{dye}}\ (1\ \text{h}) = \frac{n(\text{H}_2)_{1\ \text{h}}}{n(\text{dye}) \times t} \times 2 \;\; ; \;\; \text{TOF}_{\textbf{NiP}}\ (1\ \text{h}) = \frac{n(\text{H}_2)_{1\ \text{h}}}{n(\textbf{NiP}) \times t}$$

where TOF (1 h) = turnover frequency after 1 h of irradiation, n(H_2)$_{1\ h}$ = moles of photo-generated H_2 after 1 h of irradiation, n(dye) = moles of dye and t = time of irradiation (1 h).

$$x_u = \sum_i \frac{x_i}{n} \qquad \sigma = \sqrt{\sum_i \frac{(x_i - x_u)^2}{(n-1)}}$$

where x_u = unweighted mean value, x_i = observations, n = number of observations and σ = standard deviation.

Synthesis and characterisation.

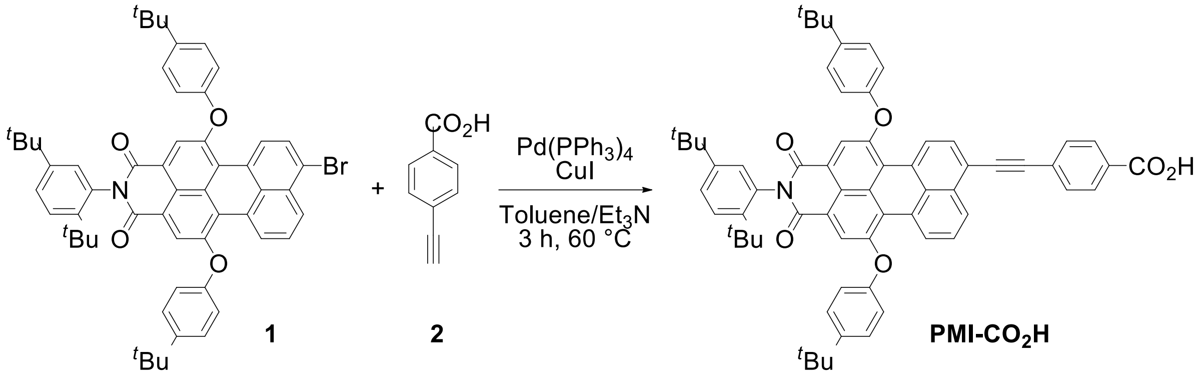

PMI-CO_2H

In a Schlenk flask, PMI-Br **1** (40 mg, 0.045 mmol, 1 eq), 4-ethynylbenzoic acid (19 mg, 0.13 mmol, 2.9 eq), copper iodide (1 mg, 0.0045 mmol, 0.1 eq) and triphenylphosphine tetrakis palladium (3 mg, 0.0023 mmol, 0.05 eq) were introduced successively under air. The Schlenk flask was purged and filled with nitrogen (5x) and a degassed mixture of dry toluene/dry triethylamine [3/1, v/v] (4 mL) was injected *via* the septum. The resulting mixture was stirred under nitrogen at 60 °C for 3 h. The crude was concentrated under reduced pressure and directly purified by chromatography on silica gel (eluent: DCM/MeOH [95/5, v/v]) and on Sephadex LH-20 column chromatography (eluent: $CHCl_3$/MeOH [6/4, v/v]). 40 mg (yield of 93%) of the desired compound **PMI-COOH** were obtained as a pink/purple solid.

^{1}H NMR (300 MHz, $CDCl_3$, 25 °C), δ (ppm): 9.38 (dd, J = 8.0 Hz, J = 0.9 Hz, 1 H), 9.29 (d, J =8.3 Hz, 1 H), 8.51 (dd, J = 8.2 Hz, J = 0.9 Hz, 1 H), 8.31 (s, 1 H), 8.29 (s, 1 H), 8.14 (d, J = 8.2 Hz, 2 H), 7.82 (d, J = 8.3 Hz, 1 H), 7.74 (d, J = 8.4 Hz, 2 H), 7.68 (t, J = 8.1 Hz, 1 H), 7.56 (d, J = 8.6 Hz, 1 H), 7.42 (m, 5 H), 7.09 (m, 4 H), 6.99 (d, J = 2.19 Hz, 1 H), 1.35 (s, 18 H), 1.30 (s, 9 H), 1.28 (s, 9 H).

^{13}C NMR (75 MHz, $CDCl_3$, 25 °C), δ ppm): 171.0, 164.2, 154.3, 154.1, 153.1, 153.0, 150.0, 147.5, 147.4, 143.8, 133.3, 132.6, 131.7, 131.6, 131.4, 130.3, 129.3, 129.1, 128.8, 128.6, 128.2, 128.1, 127.8, 127.5, 127.3, 126.6, 126.2, 123.9, 123.8, 122.6, 122.2, 122.0, 121.9, 118.8, 95.9, 91.2, 35.6, 34.5, 34.2, 31.8, 31.5, 31.2, 29.7.

HRMS (ESI+) m/z: $[M]^+$ calculated for $C_{65}H_{59}NO_6$: 949.4337; found: 949.4364. Δ = 2.9 ppm.

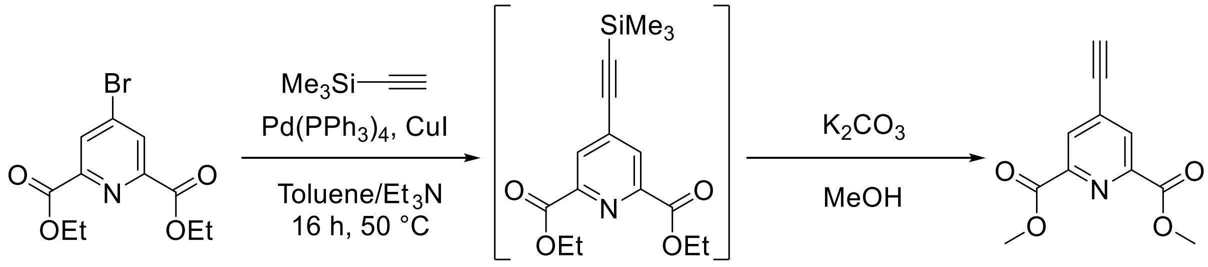

6 **7** **4**

Compound **7**

In a Schlenk flask, compound **6** (300 mg, 0.99 mmol, 1 eq), copper iodide (19 mg, 0.099 mmol, 0.1 eq) and triphenylphosphine tetrakis palladium (57 mg, 0.050 mmol, 0.05 eq) were introduced successively under air. The Schlenk flask was purged and filled with nitrogen (5x) and a degassed mixture of dry toluene/dry triethylamine [4/1, v/v] (10 mL) was injected *via* septum. Ethynyltrimethylsilane (1.4 mL, 9.93 mmol, 10 eq) was injected through the septum. The resulting mixture was stirred under nitrogen at 50 °C for 16 h. The solution was cooled to room temperature. The crude was diluted with diethyl ether, filtered on celite and washed with diethyl ether. The filtrate was transferred in a round bottom flask and then concentrated under reduced pressure. Compound **7** was used without further purification.

Compound **4**

After being under argon, methanol was injected and potassium carbonate (1.37 g, 9.93 mmol, 10 eq) was added quickly. The resulting solution was stirred at room temperature for 30 min. The solution was then diluted with water and extracted with ethyl acetate. The combined organic layer was washed with water, dried on anhydrous sodium sulfate, filtered and concentrated under reduced pressure. The crude material was then purified by flash chromatography on silica gel (DCM/petroleum spirit [0/10] to [10/0]). 134 mg (yield of 62%) of the desired compound **4** were obtained as a pale yellow solid.

^{1}H NMR (400 MHz, $CDCl_3$, 25 °C), δ (ppm): 8.31 (s, 2 H), 4.02 (s, 6 H), 3.47 (s, 1 H).

^{13}C NMR (100 MHz, $CDCl_3$, 25 °C), δ (ppm): 164.7, 148.7, 133.4, 130.4, 84.8, 79.6, 53.5.

HRMS (ESI+) m/z: $[M+H]^+$ calculated for $C_{11}H_{10}NO_4$: 220.0604; found: 220.0597. Δ = 3.4 ppm.

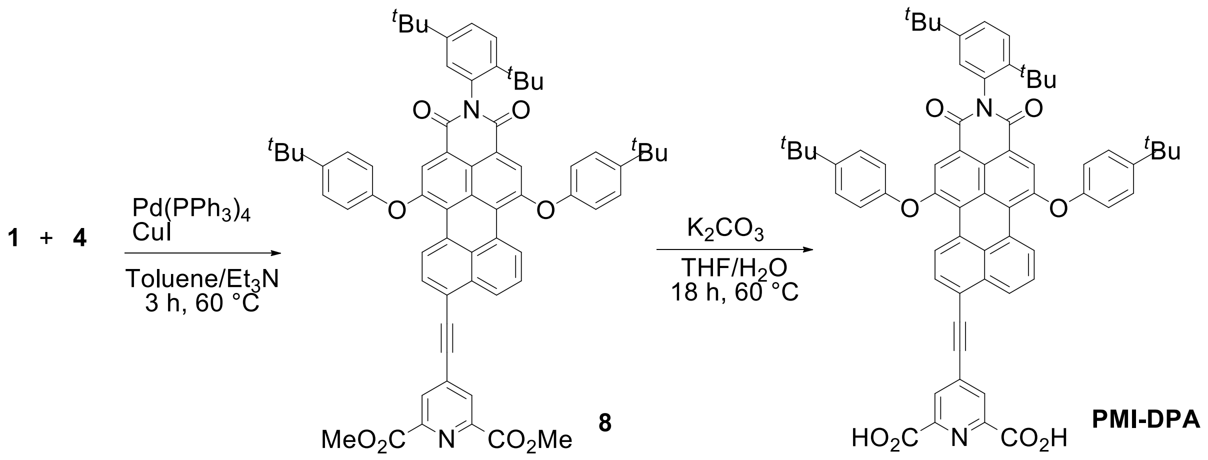

Compound **8**

In a Schlenk flask PMI-Br **1** (50 mg, 0.057 mmol, 1 eq), compound **4** (25 mg, 0.011 mmol, 2 eq), copper iodide (0.5 mg, 0.0028 mmol, 0.05 eq) and triphenylphosphine tetrakis palladium (3 mg, 0.0028 mmol, 0.05 eq) were introduced successively under air. The Schlenk flask was purged and filled with nitrogen (5x) and a degassed mixture of THF/diisopropylethylamine [4/1, v/v] (10 mL) was injected *via* septum. The resulting mixture was stirred under nitrogen at 60 °C for 18 h. An amount of compound **4** (25 mg, 0.011 mmol, 2 eq) was added and the solution was stirred for 16 h. After cooling to room temperature, the crude was concentrated under reduced pressure and directly purified by chromatography on silica gel (DCM/petroleum [0/10] to [10/0]). 19 mg (yield of 33%) of the desired compound **8** were obtained as a pink solid.

^{1}H NMR (400 MHz, $CDCl_3$, 25 °C), δ (ppm): 9.49 (dd, J = 7.9 Hz, *J* = 1.0 Hz, 1 H), 9.36 (d, *J* = 8.3 Hz, 1 H), 8.48 (m, 1 H), 8.47 (s, 2 H), 8.31 (s, 1 H), 8.29 (s, 1 H), 7.89 (d, *J* = 8.2 Hz, 1 H), 7.77 (t, *J* = 8.0 Hz, 1 H), 7.54 (d, *J* = 8.7 Hz, 1 H), 7.43 (m, 5 H), 7.09 (d, *J* = 8.8 Hz, 4 H), 6.95 (d, *J* = 2.2 Hz, 1 H), 4.06 (s, 6 H), 1.35 (s, 9 H), 1.35 (s, 9 H), 1.29 (s, 9 H), 1.26 (s, 9 H).

^{13}C NMR (100 MHz, $CDCl_3$, 25 °C), δ (ppm): 164.8, 164.1, 154.7, 154.3, 153.1, 152.9, 150.0, 148.7, 147.7, 147.6, 143.8, 134.2, 133.4, 132.5, 132.2, 131.7, 130.0, 129.6, 129.4, 129.2, 128.8, 128.3, 128.0, 127.8, 127.6, 127.5, 127.3, 127.3, 126.2, 126.2, 125.7, 123.8, 123.5, 122.7, 122.5, 120.4, 118.9, 118.9, 95.2, 92.1, 53.4, 35.5, 34.5, 34.2, 31.8, 31.5, 31.2.

HRMS (ESI+) m/z: $[M+H]^+$ calculated for $C_{67}H_{63}N_2O_8$: 1023.4579; found: $[M+H]^+$ 1023.4544. Δ = 3.4 ppm.

PMI-DPA

In a round bottom flask containing perylene **8** (31 mg, 0.031 mmol, 1 eq) and potassium carbonate (42 mg, 0.31 mmol, 10 eq) under argon atmosphere, a mixture of THF/water (4/1, v/v) was added via septum. The solution was stirred at 50 °C for 16 h. After completion of the reaction monitored by TLC, ethyl acetate and 0.1 N HCl aqueous solution was added to the mixture. The solution was extracted with ethylacetate (2x). The organic layer was dried over anhydrous sodium sulfate and filtered. The product was taken in DCM and petroleum spirit was added. The precipitate was recovered by filtration to give the title compound **PMI-DPA** with a yield of 99% (30 mg).

^{1}H NMR (300 MHz, THF-*d8*/MeOD [95/5], 25 °C), δ (ppm): 9.42 (m, 1 H), 9.34 (m, 1 H), 8.51 (d, J = 6.6 Hz, 1 H), 8.42 (s, 2 H), 8.20 (s, 2 H), 7.88 (d, J = 7.0 Hz, 1 H), 7.74 (m, 1 H), 7.50 (m, 5 H), 7.37 (d, J = 8.5 Hz, 1 H), 7.19 (m, 5 H), 1.33 (s, 9 H), 1.32 (s, 9 H), 1.26 (s, 9 H), 1.21 (s, 9 H).
^{13}C NMR (100 MHz, THF-*d8*/MeOD [95/5], 25 °C), δ (ppm): 164.9, 164.0, 155.2, 154.8, 154.1, 154.0, 150.2, 149.1, 148.1, 148.0, 144.5, 134.1, 134.0, 132.9, 132.7, 132.6, 132.3, 131.1, 130.5, 130.0, 129.7, 129.3, 128.9, 128.6, 128.2, 127.9, 126.5, 126.1, 124.1, 123.9, 123.7, 123.6, 123.3, 121.2, 119.2, 94.8, 93.0, 40.0, 34.9, 34.6, 31.9, 31.6, 31.4, 30.4.
HRMS (ESI-) m/z: $[M-H]^{-}$ calculated for $C_{65}H_{57}N_{2}O_{8}$: 993.4115; found: 993.4094. Δ = 2.1 ppm.

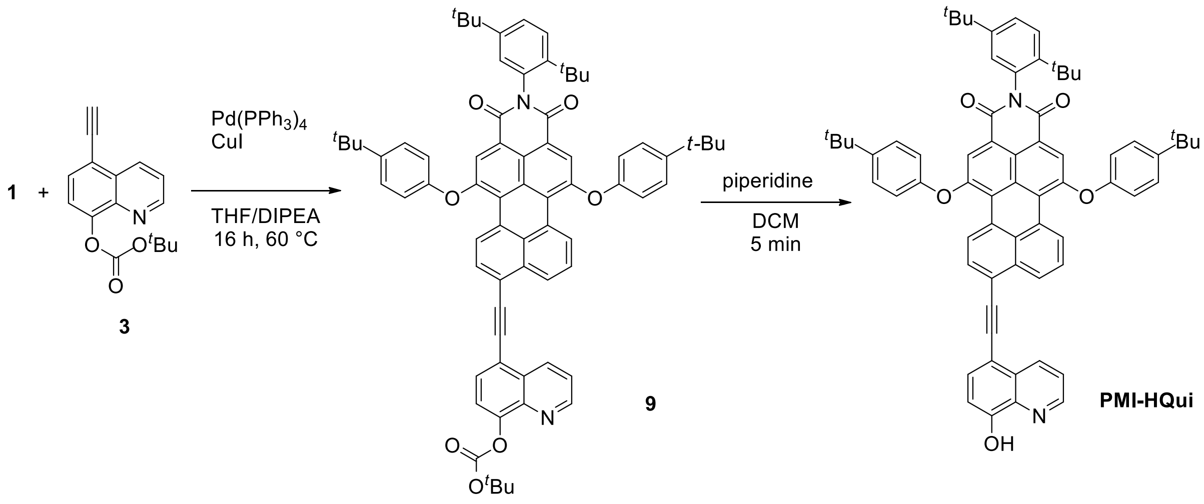

Compound **9**

In a Schlenk flask, PMI-Br **1** (60 mg, 0.068 mmol, 1 eq), compound **3** (22 mg, 0.081 mmol, 1.2 eq), copper iodide (0.6 mg, 0.0034 mmol, 0.05 eq) and triphenylphosphine tetrakis palladium (4 mg, 0.0034 mmol, 0.05 eq) were introduced successively under air. The Schlenk flask was purged and filled with nitrogen (5x) and a degassed mixture of THF/diisopropylethylamine [10/1, v/v] (10mL) was injected via septum. The resulting mixture was stirred under nitrogen at 60 °C for 16 h. An amount of compound **3** (22 mg, 0.081 mmol, 1.2 eq) was added and the solution was stirred for 6 h at the same temperature. After cooling to room temperature, the crude was concentrated under reduced pressure and directly purified by chromatography on silica gel (DCM/petroleum spirit [0/10] to [10/0]). 50 mg (yield of 69%) of the desired compound **9** were obtained as a pink-purple solid.

^{1}H NMR (300 MHz, $CDCl_3$, 25 °C), δ (ppm): 9.44 (dd, J = 7.9 Hz, J = 0.9 Hz, 1 H), 9.37 (d, J = 8.3 Hz, 1 H), 9.01 (dd, J = 4.2 Hz, J = 1.6 Hz, 1 H), 8.81 (dd, J = 8.5 Hz, J = 1.6 Hz, 1 H), 8.60 (dd, J = 8.2 Hz, J = 0.9 Hz, 1 H), 8.32 (s, 1 H), 8.31 (s, 1 H), 7.94 (dd, J = 8.1 Hz, J = 3.3 Hz, 2 H), 7.74 (t, J = 8.1 Hz, 1 H), 7.52-7.62 (m, 3 H), 7.39-7.46 (m, 5 H), 7.06-7.13 (m, 4 H), 6.94 (d, J = 2.2 Hz, 1 H), 1.62 (s, 9 H), 1.34 (s, 18 H), 1.29 (s, 9 H), 1.26 (s, 9 H).
^{13}C NMR (75 MHz, $CDCl_3$, 25 °C), δ (ppm): 164.2, 154.2, 153.1, 153.1, 151.7, 150.9, 150.0, 148.1, 147.4, 143.7, 141.2, 134.6, 133.3, 132.6, 131.7, 131.4, 130.8, 129.8, 129.4, 129.8, 128.8, 128.7, 128.2, 127.9, 127.6, 127.4, 127.2, 126.6, 126.3, 126.2, 124.0, 123.9, 122.7, 122.6, 122.3, 122.2, 122.0, 120.8, 119.1, 118.8, 118.7, 93.2, 93.1, 84.1, 35.5, 34.5, 34.2, 31.8, 31.5, 31.2, 27.7.
HRMS (ESI+) m/z: $[M+H]^+$ calculated for $C_{72}H_{69}N_2O_7$: 1073.5099; found: 1073.5098. Δ = 0.1 ppm.

PMI-HQui

In a round bottom flask under nitrogen atmosphere containing PMI **9** (38 mg, 0.035 mmol, 1 eq) in dichloromethane (5 mL) piperidine (0.1 mL, 1 mmol, 30 eq) was added. The solution was stirred at room temperature for 5 min. The solvents were evaporated and the residue taken in DCM (1 mL) and acetonitrile (20 mL) was added. The mixture was concentrated under reduced pressure (10 mL). The resulting precipitate was collected by filtration and isolated as a purple solid **PMI-HQui** with a yield of 67% (23 mg).

^{1}H NMR (400 MHz, $CDCl_3$, 25 °C), δ (ppm): 9.40 (dd, *J* = 7.9 Hz, *J* = 0.7 Hz, 1 H), 9.30 (d, *J* = 8.3 Hz, 1 H), 8.84 (dd, *J* = 4.2 Hz, *J* = 1.4 Hz, 1 H), 8.74 (dd, *J* = 8.4 Hz, *J* = 1.5 Hz, 1 H), 8.59 (dd, *J* = 8.2 Hz, *J* = 0.7 Hz, 1 H), 8.31 (s, 1 H), 8.30 (s, 1 H), 7.86 (d, *J* = 8.1 Hz, 2 H), 7.69 (t, *J* = 8.1 Hz, 1 H), 7.58 (dd, *J* = 8.5 Hz, *J* = 4.2 Hz, 1 H), 7.55 (d, *J* = 8.6 Hz, 1 H), 7.41 (m, 5 H), 7.20 (d, *J* = 8.0 Hz, 1 H), 7.09 (m, 4 H), 6.69 (d, *J* = 2.2 Hz, 1 H), 1.34 (s, 18 H), 1.30 (s, 9 H), 1.28 (s, 9 H).
^{13}C NMR (100 MHz, $CDCl_3$, 25 °C), δ (ppm): 164.3, 154.1, 154.1, 153.5, 153.3, 150.1, 148.5, 147.5, 143.9, 138.2, 135.1, 133.3, 133.0, 132.8, 131.8, 131.1, 129.5, 129.2, 129.0, 128.9, 128.5, 128.2, 128.1, 127.7, 127.5, 127.3, 126.8, 126.6, 126.3, 124.0, 123.1, 122.9, 122.2, 122.1, 118.9, 118.8, 111.3, 110.2, 94.3, 91.5, 35.7, 34.6, 34.3, 31.9, 31.6, 31.3.
HRMS (MALDI-TOF) m/z: $[M+H]^+$ calculated for $C_{67}H_{61}N_2O_5$: 973.4575; found: 973.4569. Δ = 0.7 ppm.

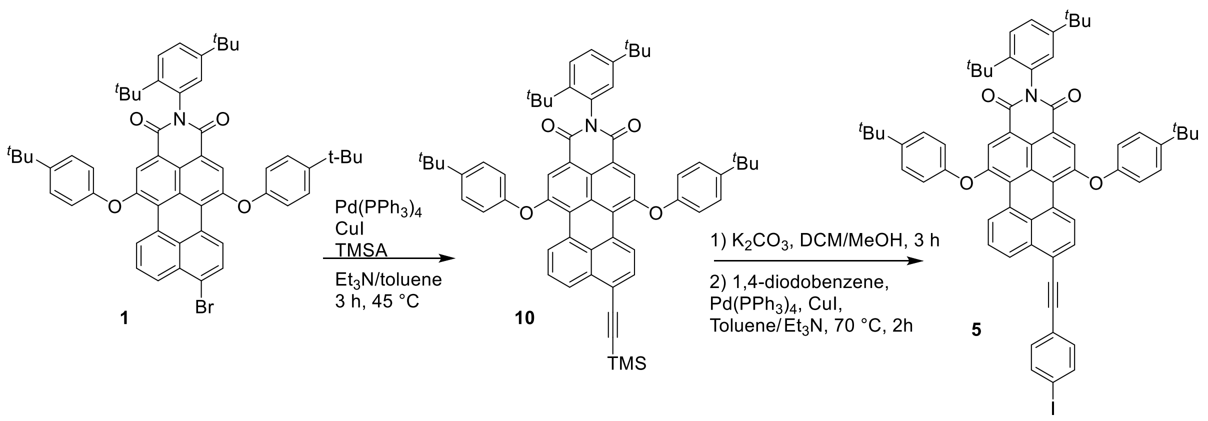

Compound **10**

The compound was synthesised following the previously published procedure.[5]

Compound **5**

The deprotection of compound **10** was performed following the previously published procedure[5] before the TMS-free PMI (200 mg, 0.24 mmol, 1 eq) was introduced in a Schlenk flask followed by 1,4-diodobenzene (1.59 g, 4.82 mmol, 20 eq), copper iodide (5 mg, 0.024 mmol, 0.1 eq) and triphenylphosphine tetrakis palladium (14 mg, 0.012 mmol, 0.05 eq). The Schlenk flask was purged and filled with nitrogen (5x) and a degassed mixture of dry toluene/dry triethylamine [2/1, v/v] (9 mL) was injected via septum. The resulting mixture was stirred under nitrogen at 70 °C for 2 h. The crude was concentrated under reduced pressure and directly purified by chromatography on silica gel (eluent: petroleum spirit/DCM [8/2, v/v] to petroleum spirit/DCM [4/6, v/v]). 202 mg (yield of 81%) of the desired compound **5** were obtained as an orange-red solid.

^{1}H NMR (300 MHz, $CDCl_3$, 25 °C), δ (ppm): 9.29 (dd, *J* = 7.9 Hz, *J* = 1.0 Hz, 1 H), 9.16 (d, *J* = 8.3 Hz, 1 H), 8.44 (dd, *J* = 8.2 Hz, *J* = 1.0 Hz, 1 H), 8.32 (s, 1 H), 8.29 (s, 1 H), 7.68-7.74 (m, 3 H), 7.55-7.63 (m, 2 H), 7.45-7.48 (m, 1 H), 7.42 (m, 4 H), 7.35 (m, 2 H), 7.03-7.14 (m, 5 H), 1.37 (s, 9 H), 1.36 (s, 9 H), 1.33 (s, 9 H), 1.32 (s, 9 H).

^{13}C NMR (75 MHz, $CDCl_3$, 25 °C), δ (ppm): 164.2, 154.1, 154.0, 153.1, 153.0, 150.1, 147.5, 147.4, 143.8, 137.7, 133.2, 133.1, 132.7, 131.5, 131.0, 129.1, 128.9, 128.9, 128.4, 128.2, 127.0, 127.8, 127.5, 127.3, 126.5, 126.3, 126.2, 123.9, 123.7, 122.6, 122.6, 122.3, 122.0, 121.9, 118.9, 118.9, 96.0, 94.8, 89.4, 35.6, 34.5, 34.3, 31.9, 31.5, 31.3.

HRMS (ESI +) m/z: $[M]^+$ calculated for $C_{64}H_{58}O_4NI$: 1031.3411; found: 1031.3419. Δ = 0.8 ppm.

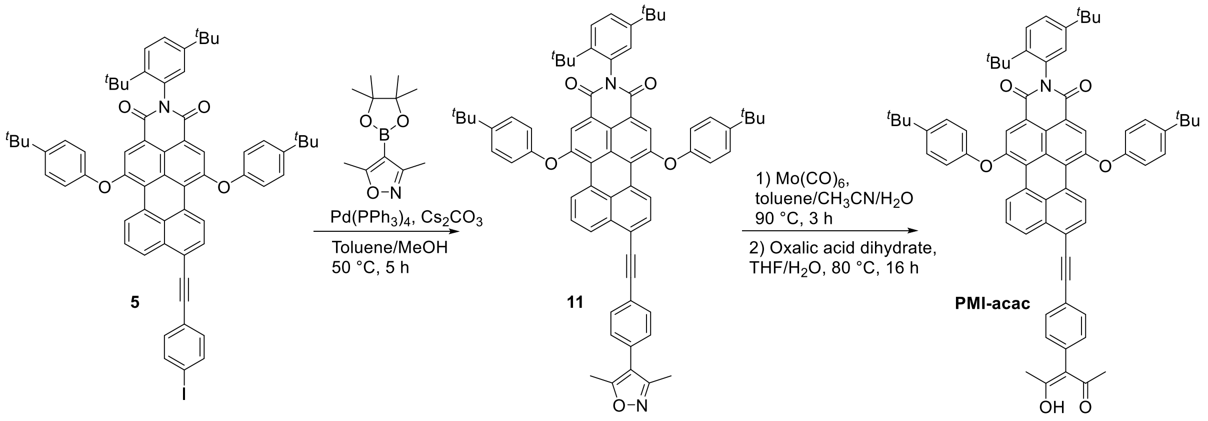

Compound **11**

PMI **5** (50 mg, 0.048 mmol, 1 eq), dimethylisoxasol boronic acid (20 mg, 0.145 mmol, 3 eq), triphenylphosphine tetrakis palladium (6 mg, 0.0048 mmol, 0.1 eq) and cesium carbonate (79 mg, 0.24 mmol, 5 eq) were introduced under air in a sealed tube. The degassed mixture toluene/methanol (1/1, v/v) (10 mL) was injected via septum. The solution was stirred at 50 °C for 5 h. After cooling to room temperature, the crude was concentrated under reduced pressure and purified by flash chromatography on silica gel (eluent: petroleum spirit/dichloromethane [10/0] to [0/10]). The desired product **11** was obtained as a pink solid with a yield of 99% (45 mg).

^{1}H NMR (300 MHz, CDCl$_3$, 25 °C), δ (ppm): 9.40 (dd, J = 7.9 Hz, J = 1.0 Hz, 1 H), 9.30 (d, J = 8.3 Hz, 1 H), 8.55 (dd, J = 8.2 Hz, J = 1.0 Hz, 1 H), 8.31 (s, 1 H), 8.29 (s, 1 H), 7.83 (d, J = 8.3 Hz, 1 H), 7.75 (d, J = 8.4 Hz, 2 H), 7.72 (t, J = 8.1 Hz, 1 H), 7.55 (d, J = 8.6 Hz, 1 H), 7.42 (m, 5 H), 7.32 (m, 2 H), 7.09 (m, 4 H), 6.96 (d, J = 2.2 Hz, 1 H), 2.46 (s, 3 H), 2.33 (s, 3 H), 1.34 (s, 18 H), 1.29 (s, 9 H), 1.27 (s, 9 H).

^{13}C NMR (75 MHz, CDCl$_3$, 25 °C), δ (ppm): 165.5, 164.2, 158.5, 154.1, 154.0, 153.2, 153.1, 150.0, 147.4, 143.7, 133.3, 132.6, 132.2, 131.6, 131.1, 131.0, 129.3, 129.1, 128.8, 128.4, 128.1, 127.9, 127.5, 127.4, 127.2, 126.7, 126.4, 124.0, 123.8, 122.7, 122.6, 122.3, 122.1, 121.9, 118.8, 118.8, 116.2, 96.4, 88.7, 46.0, 35.6, 34.5, 34.2, 31.8, 31.5, 31.2.

HRMS (MALDI-TOF) m/z: $[M]^{+\cdot}$ calculated for $C_{69}H_{64}O_5N_2$: 1000.4810; found: 1000.4774. Δ = 3.6 ppm.

PMI-Acac

In a round bottom flask under nitrogen atmosphere containing PMI **11** (46 mg, 0.046 mmol, 1 eq) in a solution toluene/acetonitrile/water [4/3/1, v/v/v] (8 mL), molybdenumhexacarbonyl complex (24 mg, 0.092 mmol, 2 eq) was added. The solution was stirred at 90 °C for 3 h. After cooling to room temperature, the solvents were evaporated and the residue taken in DCM. The solution was washed with water (2x), dried over anhydrous sodium sulfate, filtered and concentrated under reduced pressure. NMR spectrum of the crude material reveals a mixture of ketoenamine (main) and ketoenol. The crude was taken in a mixture THF/water [8/2, v/v] (10 mL) under argon and oxalic acid dihydrate (29 mg, 0.23 mmol, 5 eq) was added. The solution was stirred at 80 °C for 16 h. After cooling to room temperature, the crude was concentrated under reduced pressure. DCM was added and the solution was washed with water (2x), dried over anhydrous sodium sulfate, filtered and concentrated under reduced pressure. Purification by preparative HPLC affords the wanted compound **PMI-Acac** with a yield of 78% (36 mg) as a pink solid.

^{1}H NMR (300 MHz, $CDCl_3$, 25 °C), δ (ppm): 16.70 (s, 1 H), 9.41 (dd, *J* = 7.9 Hz, *J* = 1.0 Hz, 1 H), 9.31 (d, *J* = 8.2 Hz, 1 H), 8.56 (dd, *J* = 8.2 Hz, *J* = 1.0 Hz, 1 H), 8.31 (s, 1 H), 8.29 (s, 1 H), 7.83 (d, *J* = 8.3 Hz, 1 H), 7.70 (m, 3 H), 7.55 (d, *J* = 8.6 Hz, 1 H), 7.42 (m, 5 H), 7.25 (m, 2 H), 7.09 (m, 4 H), 6.96 (d, *J* = 2.2 Hz, 1 H), 1.94 (s, 6 H), 1.34 (s, 18 H), 1.30 (s, 9 H), 1.27 (s, 9 H).

^{13}C NMR (75 MHz, $CDCl_3$, 25 °C), δ (ppm): 190.8, 164.2, 154.1, 154.0, 153.2, 153.1, 150.0, 147.4, 147.4, 143.7, 137.5, 133.3, 132.6, 132.2, 131.6, 131.4, 131.1, 129.3, 129.1, 128.8, 128.4, 128.3, 128.1, 127.9, 127.4, 127.4, 127.2, 126.7, 126.4, 126.2, 124.0, 123.8, 122.7, 122.6, 122.4, 122.1, 121.9, 118.8, 114.7, 96.4, 88.5, 35.6, 34.5, 34.2, 31.8, 31.5, 31.2, 24.2.

HRMS (ESI +) m/z: $[M]^+$ calculated for $C_{69}H_{65}NO_6$: 1003.4806; found: 1003.4851. Δ = 4.5 ppm.

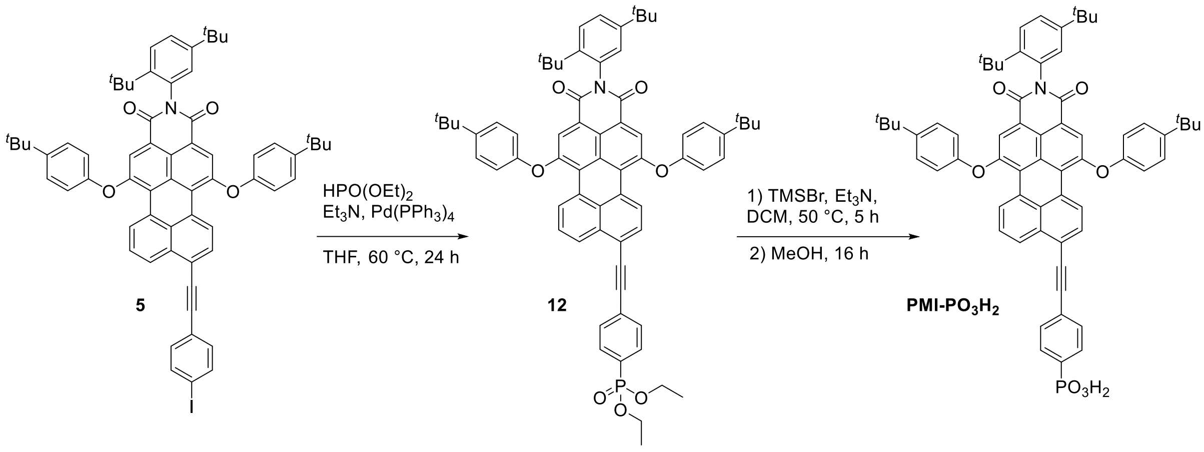

Compound **12**

In a Schlenk flask PMI **5** (50 mg, 0.048 mmol, 1 eq) and triphenylphosphine tetrakis palladium (6 mg, 0.0048 mmol, 0.1 eq) were introduced successively under air. The Schlenk flask was purged and filled with nitrogen (5x). Degassed and dry THF (5 mL) was injected via septum. Triethylamine (20 µL, 0.14 mmol, 3.00 eq) and diethylphosphite (20 µL, 0.14 mmol, 3 eq) were then injected via septum. The resulting mixture was stirred under nitrogen at 60 °C for 24 h. The crude was concentrated under reduced pressure and directly purified by chromatography on silica gel (eluent: DCM to DCM/MeOH [99/1, v/v]). 47 mg (yield of 93%) of the desired compound **12** were obtained as a pink solid.

^{1}H NMR (300 MHz, $CDCl_3$, 25 °C), δ (ppm): 9.39 (dd, J = 7.9 Hz, J = 1.0 Hz, 1 H), 9.30 (d, J = 8.3 Hz, 1 H), 8.51 (dd, J = 8.2 Hz, J = 1.0 Hz, 1 H), 8.30 (s, 1 H), 8.28 (s, 1 H), 7.90-7.91 (m, 3 H), 7.72-7.68 (m, 2 H), 7.69 (t, J = 8.1 Hz, 1 H), 7.55 (d, J = 8.6 Hz, 1 H), 7.42 (m, 5 H), 7.08 (m, 4 H), 6.96 (d, J = 2.2 Hz, 1 H), 4.05-4.27 (m, 4 H), 1.36 (t, J = 7.2 Hz, 6 H), 1.34 (s, 18 H), 1.29 (s, 9 H), 1.26 (s, 9 H).

^{13}C NMR (75 MHz, $CDCl_3$, 25 °C), δ (ppm): 164.1, 154.3, 154.1, 150.0, 147.5, 147.4, 143.7, 133.3, 132.6, 131.9, 131.8, 131.7, 131.6, 131.5, 131.4, 129.7, 129.3, 129.1, 128.8, 128.7, 122.2, 128.1, 127.8, 127.5, 127.4, 127.2, 126.6, 126.2, 124.0, 123.8, 122.6, 122.2, 122.0, 122.0, 118.8, 118.8, 95.7, 90.4, 62.4, 62.3, 35.5, 34.5, 34.2, 31.8, 31.5, 31.2, 29.7, 16.4, 16.3.

^{31}P NMR (162 MHz, $CDCl_3$, 25 °C), δ (ppm): 17.8.

HRMS (MALDI-TOF) m/z: $[M]^{+\cdot}$ calculated for $C_{68}H_{68}NO_7P$: 1041.4728; found: 1041.4750. Δ = 2.1 ppm.

PMI-PO_3H_2

In a round bottom flask containing PMI **12** (47 mg, 0.045 mmol, 1 eq) under argon atmosphere dry DCM (5 mL), triethylamine (0.4 mL) and TMS-Br (0.1 mL) were added successively. The solution was stirred for 16 h at room temperature. The mixture was then concentrated to dryness and put under argon atmosphere. Methanol was added (15 mL) and the solution was stirred for 16 h at room temperature. DCM was then added and the organic layer was washed with aqueous HCl (1 N) (3x). Evaporation to dryness give the sensitizer **PMI-PO_3H_2** with a yield of 99% (45 mg).

^{1}H NMR (300 MHz, $CDCl_3$/MeOD [9/1], 25 °C), δ (ppm): 9.04 (m, 1 H), 8.96 (m, 1 H), 8.51 (m, 1 H), 8.12 (s, 1 H), 8.10 (s, 1 H), 7.70-7.90 (m, 3 H), 7.51 (m, 2 H), 7.46 (d, J = 8.6 Hz, 2 H), 7.27-7.37 (m, 5 H), 7.01 (m, 1 H), 6.96 (m, 4 H), 1.28 (s, 9 H), 1.26 (s, 9 H), 1.18 (s, 9 H), 1.15 (s, 9 H).

^{13}C NMR (75 MHz, $CDCl_3$/MeOD [9/1], 25 °C), δ (ppm): 164.1, 164.0, 154.0, 153.8, 153.2, 153.2, 150.1, 147.5, 147.4, 143.7, 133.1, 132.5, 131.9, 131.8, 131.6, 131.2, 129.8, 129.0, 128.9, 128.8, 128.7, 126.6, 128.3, 128.0, 127.8, 127.3, 126.7, 126.4, 126.1, 123.8, 123.8, 122.7, 122.3, 122.2, 121.9, 118.7, 118.7, 96.3, 89.9, 35.6, 34.5, 34.3, 31.8, 31.5, 31.1.

HRMS (ESI-) m/z: $[M-H]^-$ calculated for $C_{64}H_{59}O_7NP$: 984.4029; found: 984.4060. Δ = 3.1 ppm.

Supporting Tables

Table S1. Summary of Gibbs energies of the different PMI derivatives towards electron injection (ΔG_{inj}) and charge regeneration (ΔG_{reg}) by the electron donor.

Dye	ΔG_{inj} (4.5)[a] (eV)	ΔG_{inj} (7.0)[a] (eV)	ΔG_{inj} (8.5)[a] (eV)	ΔG_{reg} (AA)[b] (eV)	ΔG_{reg} (TEOA)[b] (eV)
**PMI-CO$_2$H**	−0.22	−0.07	+0.02	−1.24	−0.62
**PMI-Acac**	−0.33	−0.18	−0.09	−1.14	−0.52
**PMI-PO$_3$H$_2$**	−0.23	−0.08	+0.01	−1.23	−0.61
**PMI-HQui**	−0.46	−0.31	−0.22	−1.00	−0.38
**PMI-DPA**	−0.20	−0.05	+0.04	−1.29	−0.67

[a]ΔG_{inj} (pH) were calculated from the equation: ΔG_{inj} (pH) = $E(S^+/S^*)$-$E_{CB}(TiO_2)$ with $E_{CB}(TiO_2)$ = −0.55, −0.70 and −0.79 V vs. NHE at pH 4.5, 7.0 and 8.5 respectively. [b]ΔG_{reg} (ED) were calculated from the equation: ΔG_{reg} (ED) = $-(E(S^+/S)-E_{ox}(ED))$ with E_{ox}(AA) = 0.20 V *vs* NHE and E_{ox}(TEOA) = 0.82 V *vs* NHE.

Table S2. Photovoltaic performance of DSC devices sensitised with the PMI dyes and recorded under 1 Sun (AM1.5G) at room temperature in ACN using I_3^-/I^- as a redox mediator.

Dye	J_{SC} (mA cm^{-2})	V_{OC} (mV)	*FF* (%)	η (%)
**PMI-CO$_2$H**	2.87 ± 0.20	440 ± 5	72 ± 1	0.90 ± 0.10
**PMI-Acac**	0.53 ± 0.05	390 ± 10	68 ± 1	0.14 ± 0.10
**PMI-PO$_3$H$_2$**	4.20 ± 0.30	480 ± 20	70 ± 2	1.42 ± 0.10
**PMI-HQui**	5.60 ± 0.50	460 ± 10	71 ± 2	1.80 ± 0.20
**PMI-DPA**	3.28 ± 0.20	430 ± 5	71 ± 2	1.00 ± 0.01

Table S3. PMI dyes loaded (n_{PMI}) on TiO_2|Pt nanoparticles.

Dye	Dye immobilised (%)[a]	n_{PMI} on 2 mg of TiO_2|Pt (nmol)
**PMI-CO$_2$H**	66.5 ± 2.0	26.6
**PMI-Acac**	81.6 ± 3.8	32.4
**PMI-PO$_3$H$_2$**	96.9 ± 1.4	38.6
**PMI-HQui**	86.6 ± 1.1	34.6
**PMI-DPA**	84.1 ± 2.1	33.6

[a] calculated from the n_{PMI}/n_{tot} ratio, with n_{tot} = number of mole of PMI in the dyeing solution

Table S4. Comparison of photocatalytic activity of PMI|TiO_2|Pt at pH 4.5 (AA buffer, 0.1 M).

Entry	Dye[a]	$n(H_2)$ / µmol (1 h)	TOF_{PMI} / h^{-1} (1 h)[b]	$n(H_2)$ / µmol (24 h)	TON_{PMI} (24 h)[b]
1	**PMI-CO_2H**	2.86 ± 0.31	344 ± 38	53.7 ± 6.2	6461 ± 749
2	**PMI-Acac**	1.14 ± 0.12	112 ± 12	21.7 ± 2.2	2146 ± 203
3	**PMI-PO_3H_2**	2.52 ± 0.33	210 ± 27	42.5 ± 6.3	3546 ± 523
4	**PMI-HQui**	5.05 ± 0.78	467 ± 72	53.3 ± 5.9	4928 ± 549
5	**PMI-DPA**	3.20 ± 0.62	305 ± 59	41.4 ± 2.9	3943 ± 394
6	**PMI-CO_2H|TiO_2|NiP**	0.24 ± 0.03	28.6 ± 4.0	1.62 ± 0.25	169.9 ± 25.9

[a]Experiments were performed in triplicate. Conditions: 1.25 mg PMI|TiO_2|Pt, 3 mL of AA SED solution (0.1 M, pH 4.5). Samples were kept at 25 °C and irradiated with UV-filtered solar light (100 mW cm^{-2}, AM 1.5G, λ > 420 nm). [b]TOF and TON were calculated from $n(H_2)$ taking into account the individual loadings of the PMI dyes.

Table S5. Comparison of TOFs of PMI|TiO_2|Pt at pH 4.5, 7.0 and 8.5.

Dye[a]	pH	TOF_{PMI} / h^{-1} (1 h)	TOF_{PMI} / h^{-1} (24 h)	*Retained activity*
**PMI-CO_2H**	4.5	344.0	269.2	78%
	7.0	59.2	19.2	33%
	8.5	58.6	20.4	35%
**PMI-Acac**	4.5	112.0	89.4	80%
	7.0	10.9	5.5	51%
	8.5	23.4	12.3	52%
**PMI-PO_3H_2**	4.5	210.0	147.8	70%
	7.0	27.4	12.6	46%
	8.5	60.9	29.5	48%
**PMI-HQui**	4.5	497.0	205.3	44%
	7.0	25.6	9.7	38%
	8.5	26.4	10.9	41%
**PMI-DPA**	4.5	305.0	164.3	54%
	7.0	27.5	15.3	55%
	8.5	32.8	18.5	56%

Table S6. Comparison of photocatalytic activity of PMI|TiO_2|Pt at pH 7 (TEOA buffer, 0.1 M).

Entry	Dye[a]	$n(H_2)$ / µmol (1 h)	TOF_{PMI} / h^{-1} (1 h)[b]	$n(H_2)$ / µmol (24 h)	TON_{PMI} (24 h)[b]
7	**PMI-CO_2H**	0.49 ± 0.05	59.2 ± 5.9	3.9 ± 0.5	471 ± 63
8	**PMI-Acac**	0.11 ± 0.01	10.9 ± 1.0	1.3 ± 0.1	133 ± 13
9	**PMI-PO_3H_2**	0.33 ± 0.03	27.4 ± 2.7	3.6 ± 0.4	303 ± 30
10	**PMI-HQui**	0.28 ± 0.03	25.6 ± 2.6	2.5 ± 0.5	232 ± 26
11	**PMI-DPA**	0.29 ± 0.03	27.5 ± 2.7	3.8 ± 0.2	366 ± 37

[a]Experiments were performed in triplicate. Conditions: 1.25 mg PMI|TiO_2|Pt, 3 mL of TEOA SED solution (0.1 M, pH 7). Samples were kept at 25 °C and irradiated with UV-filtered solar light (100 mW cm^{-2}, AM 1.5G, λ > 420 nm). [b]TOF and TON were calculated from $n(H_2)$ taking into account the individual loadings of the PMI dyes.

Table S7. Comparison of photocatalytic activity of PMI|TiO_2|Pt at pH 8.5 (TEOA buffer, 0.1 M).

Entry	Dye[a]	$n(H_2)$ / µmol (1 h)	TOF_{PMI} / h^{-1} (1 h)[b]	$n(H_2)$ / µmol (24 h)	TON_{PMI} (24 h)[b]
12	**PMI-CO_2H**	0.48 ± 0.12	58.6 ± 14.5	4.1 ± 1.4	490 ± 170
13	**PMI-Acac**	0.24 ± 0.03	23.4± 3.1	3.0 ± 0.7	294 ± 67
14	**PMI-PO_3H_2**	0.73 ± 0.07	60.9 ± 6.1	8.5 ± 1.3	708 ± 107
15	**PMI-HQui**	0.29 ± 0.03	26.4 ± 2.8	2.8 ± 0.4	262 ± 36
16	**PMI-DPA**	0.34 ± 0.03	32.8 ± 3.3	4.7 ± 0.7	444 ± 62

[a]Experiments were performed in triplicate. Conditions: 1.25 mg PMI|TiO_2|Pt, 3 mL of TEOA SED solution (0.1 M, pH 8.5). Samples were kept at 25 °C and irradiated with UV-filtered solar light (100 mW cm^{-2}, AM 1.5G, λ > 420 nm). [b]TOF and TON were calculated from $n(H_2)$ taking into account the individual loadings of the PMI dyes.

Supporting Figures

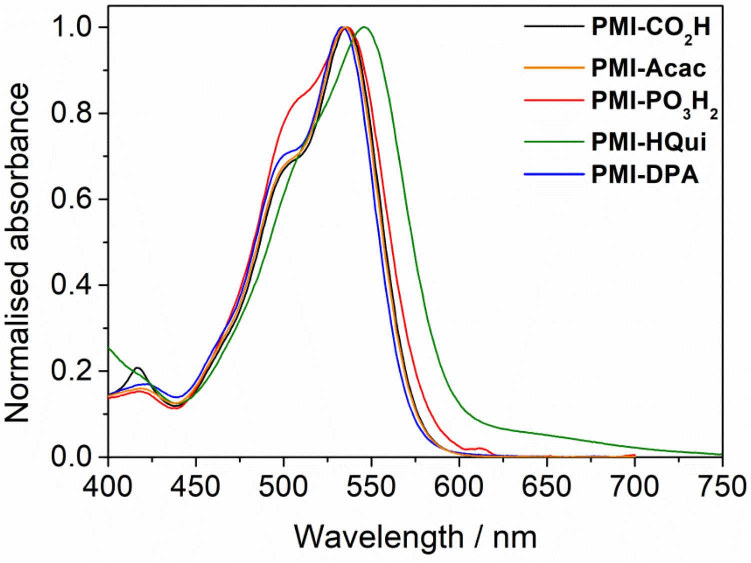

Figure S1. Room temperature UV-vis absorption spectra of the PMI dyes in DMF solution.

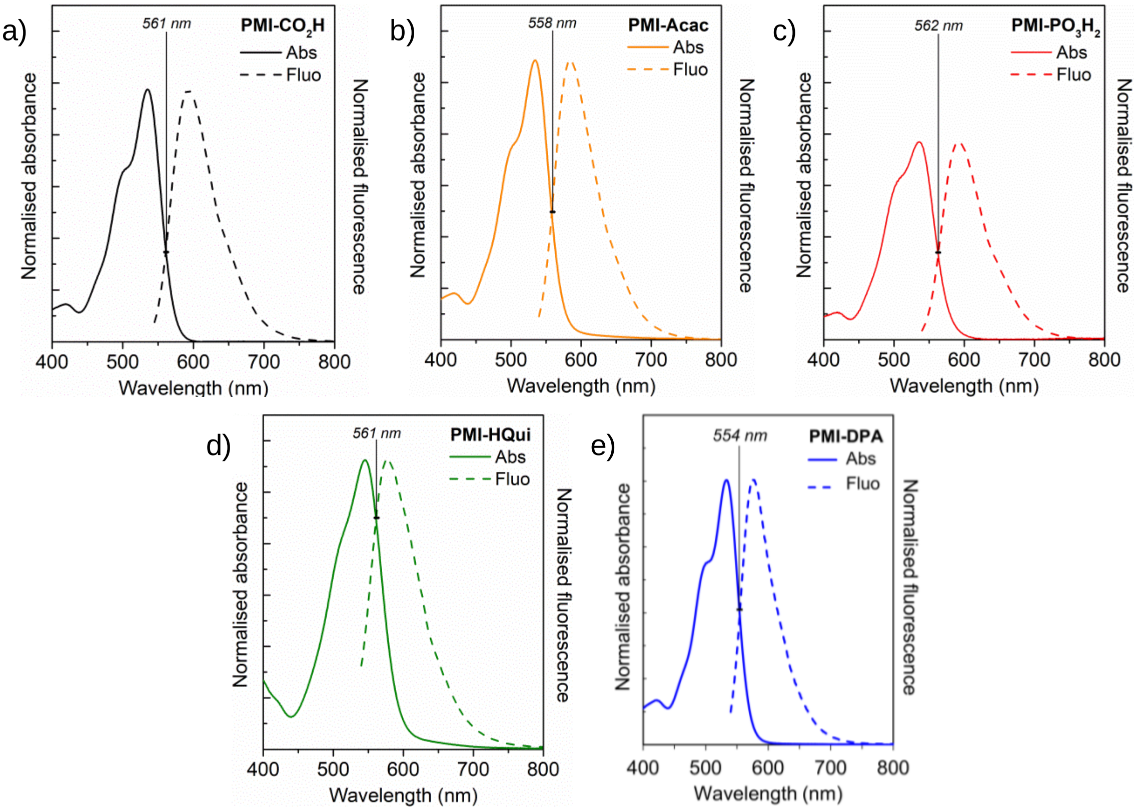

Figure S2. Normalised absorption and emission spectra of a) **PMI-CO$_2$H**, b) **PMI-Acac**, c) **PMI-PO$_3$H$_2$**, d) **PMI-HQui** and e) **PMI-DPA** in DMF (diluted solution, room temperature).

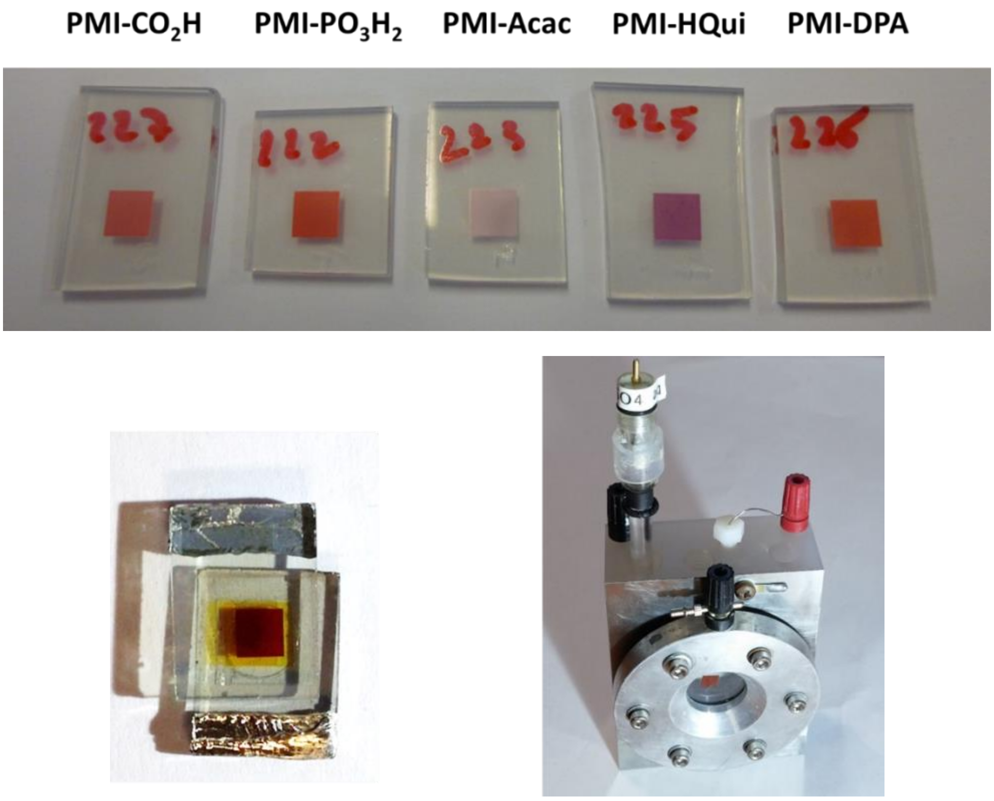

Figure S3. Pictures of the dyed TiO_2 films (S = 0.25 cm^2) with each PMI sensitiser (up), of a DSC (down left) and of the cell used for the chopped light measurements (down, right).

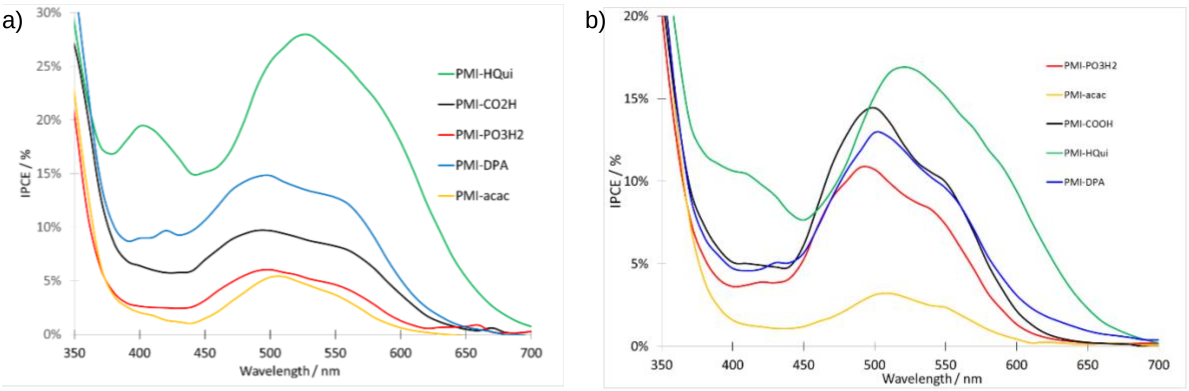

Figure S4. IPCE spectra of the PMI-sensitised DSCs using a) the acetonitrile based electrolyte (50 mM I_2, 0.1 M LiI and 0.6 M 1-ethyl-2,3-dimethylimidazolium iodide) and b) aqueous electrolyte (20 mM I_2, 4 M KI in water saturated with chenodeoxycholic acid).

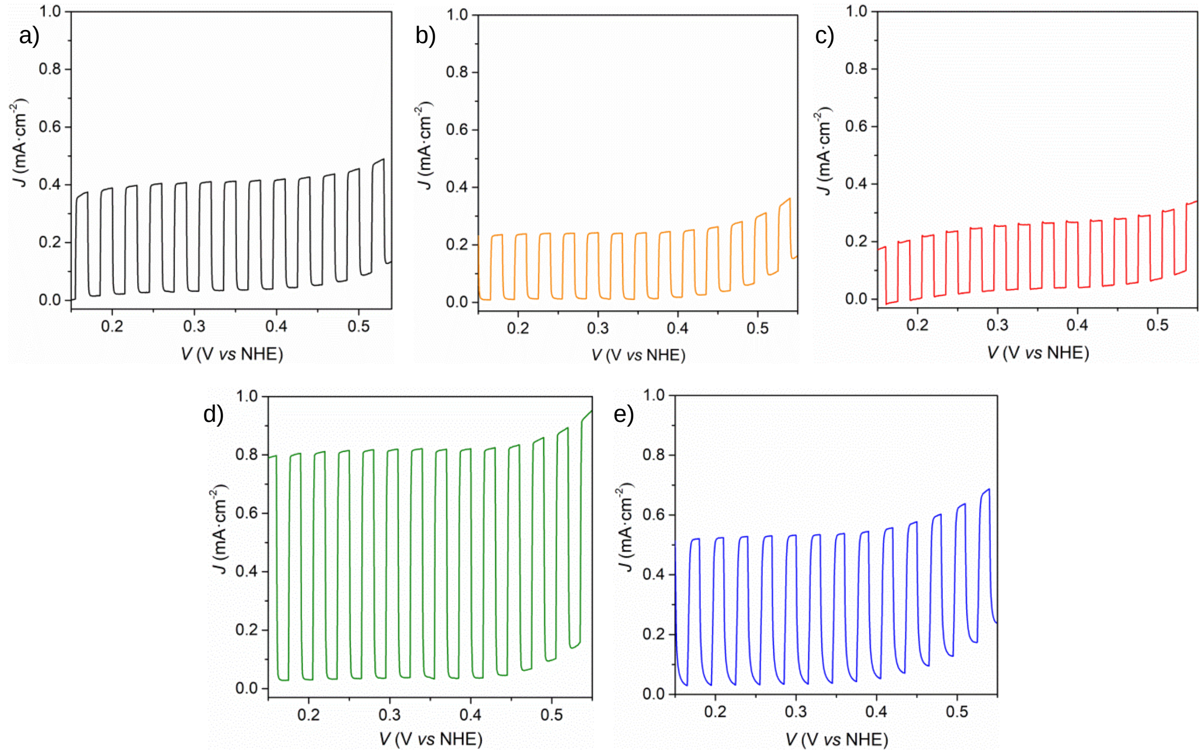

Figure S5. Linear sweep voltammogram of the TiO_2 electrodes sensitised with a) **PMI-CO_2H**, b) **PMI-Acac**, c) **PMI-PO_3H_2**, d) **PMI-HQui** and e) **PMI-DPA** evaluated in H_2O. Conditions: room temperature, [AA] = 0.1 M, pH 4.5, 5 mV s^{-1}, white light 2 W m^{-2}.

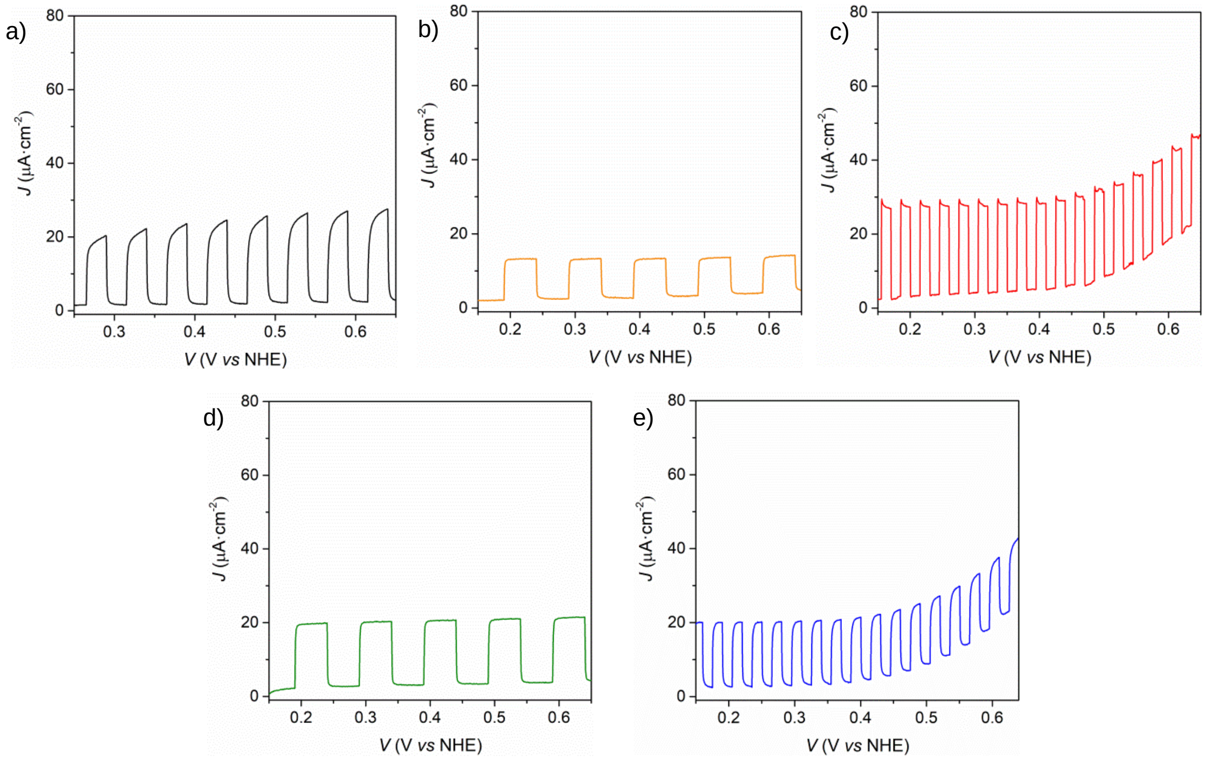

Figure S6. Linear sweep voltammograms of the TiO_2 electrodes sensitised with a) **PMI-CO_2H**, b) **PMI-Acac**, c) **PMI-PO_3H_2**, d) **PMI-HQui** and e) **PMI-DPA** evaluated in H_2O. Conditions: room temperature, [TEOA] = 0.1 M, pH 8.5, 5 mV s^{-1}, white light 2 W m^{-2}.

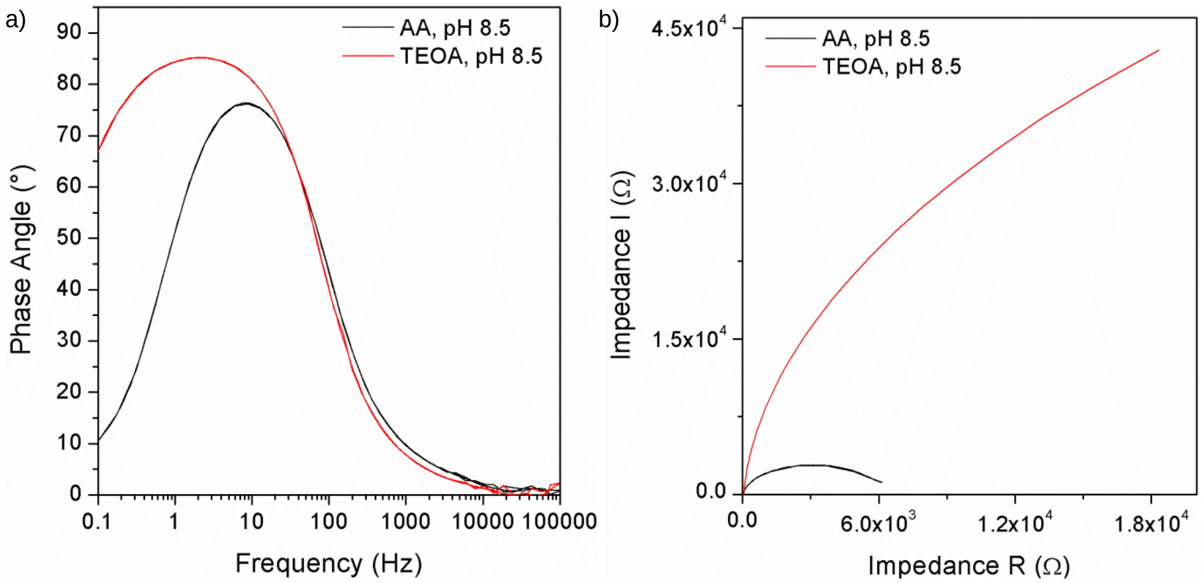

Figure S7. Electrochemical impedance spectroscopy a) Bode plots and b) Nyquist plot measurements obtained with **PMI-CO₂H** sensitised TiO_2 electrodes evaluated in H_2O using TEOA (red line) or AA (black line) as electron donors. Conditions: room temperature, open-circuit potential, [TEOA] = [AA] = 0.1 M, white light 2 W m^{-2}.

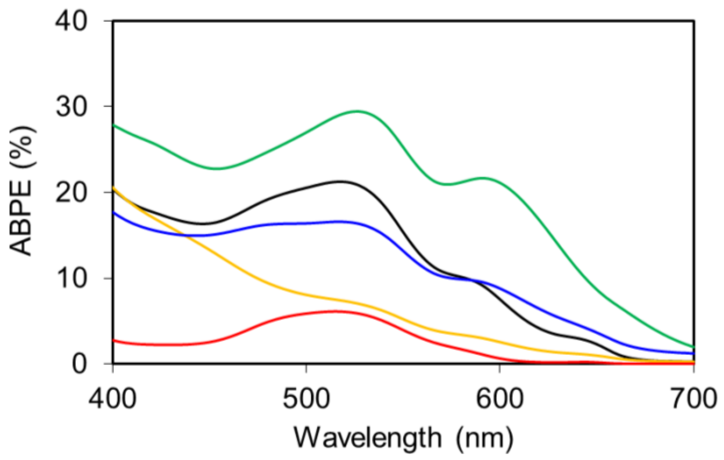

Figure S8. ABCE spectra of the TiO_2 photoelectrochemical cells dyed with **PMI-CO_2H** (black), **PMI-Acac** (yellow), **PMI-PO_3H_2** (red), **PMI-HQui** (green) and **PMI-DPA** (blue traces), using AA (0.1 M in water) as a ED at E_{app} = +0.25 V *vs* NHE and recorded at room temperature.

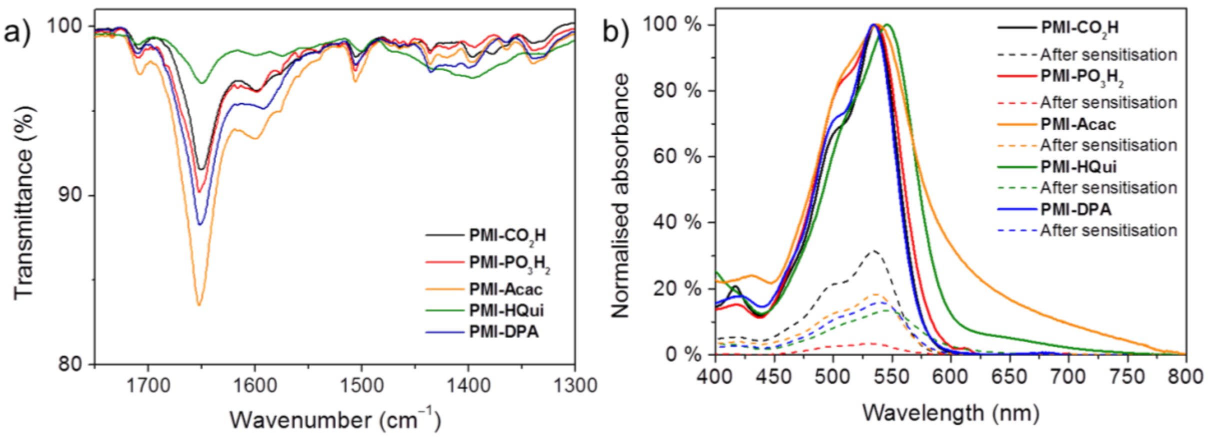

Figure S9. Dyes' a) IR spectra on TiO_2 nanoparticles and b) UV-vis spectra of the sensitising bath before and after dye loading on platinised TiO_2 nanoparticles.

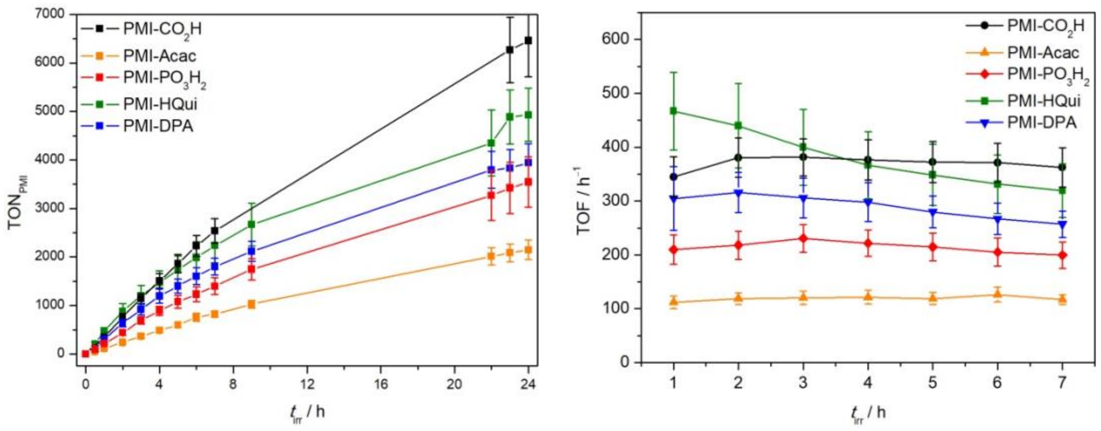

Figure S10. Comparison of photocatalytic activity of PMI|TiO_2|Pt. Conditions: 1.25 mg PMI|TiO_2|Pt, 3 mL AA buffer solution (0.1 M, pH 4.5), UV-filtered simulated solar light irradiation (100 mW cm^{-2}, AM 1.5G, λ > 420 nm) at 25 °C (see Table S4).

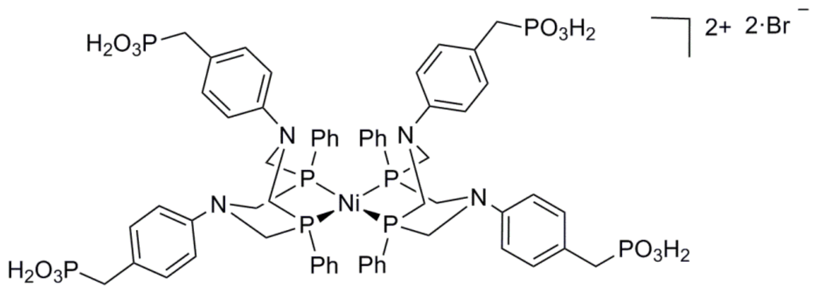

Figure S11. Structure of the molecular catalyst **NiP**.

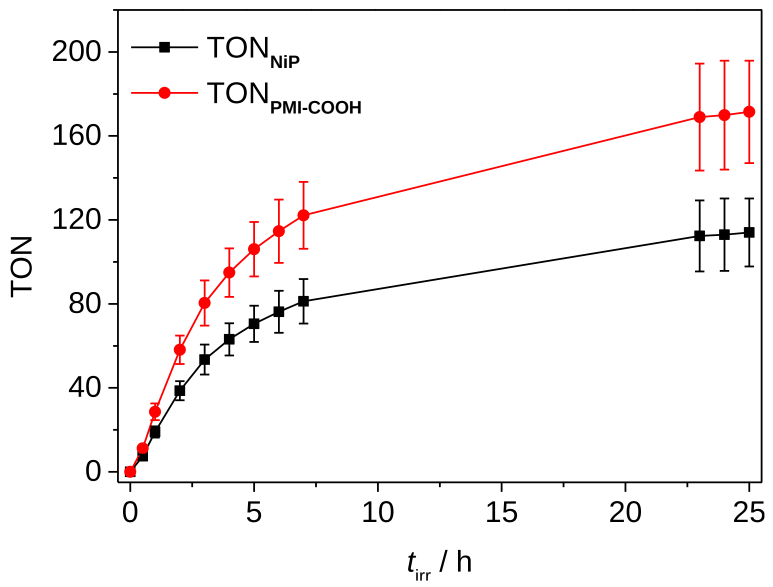

Figure S12. Photocatalytic activity of **PMI-CO₂H**|TiO₂|**NiP**. Conditions: 1.25 mg PMI|TiO₂, 12.5 nmol **NiP**, 3 mL AA solution (0.1 M, pH 4.5), UV-filtered simulated solar light irradiation (100 mW cm^{-2}, AM 1.5G, λ > 420 nm) at 25 °C (see Table S4).

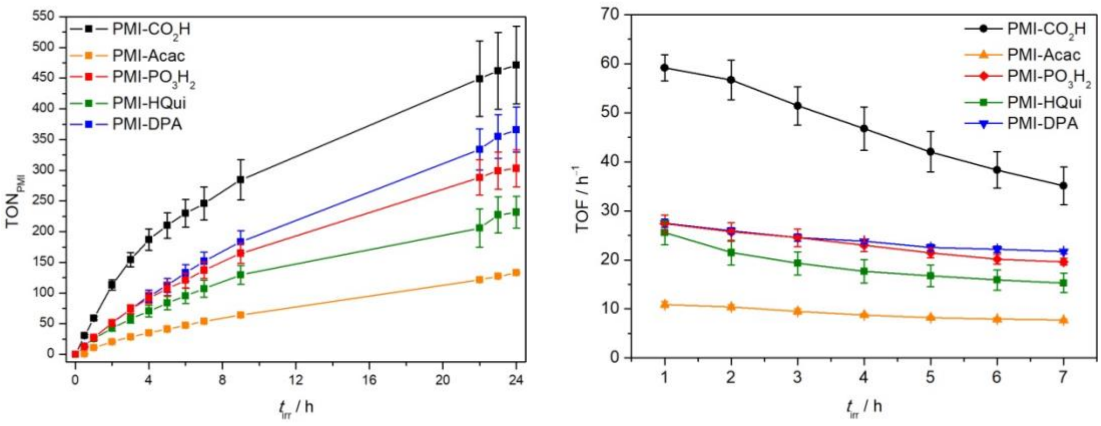

Figure S13. Comparison of photocatalytic activity of PMI|TiO₂|Pt. Conditions: 1.25 mg PMI|TiO₂|Pt, 3 mL TEOA buffer solution (0.1 M, pH 7), UV-filtered simulated solar light irradiation (100 mW cm^{-2}, AM 1.5G, λ > 420 nm) at 25 °C (see Table S6).

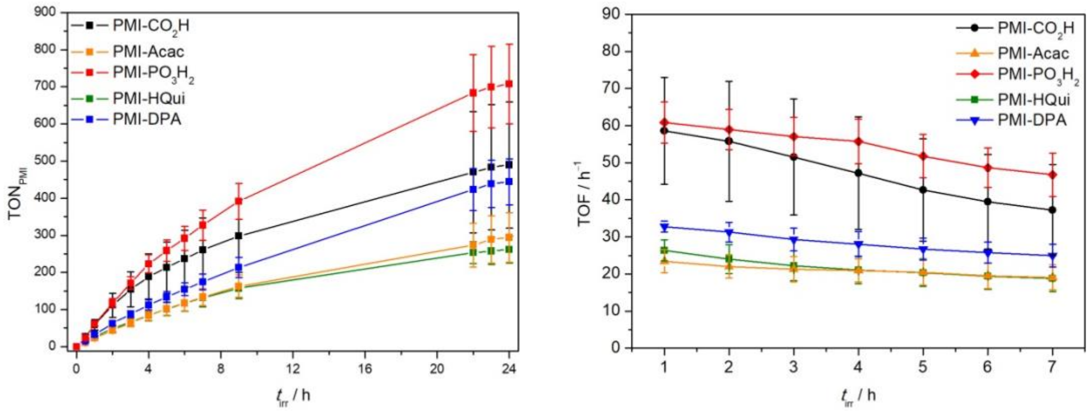

Figure S14. Comparison of photocatalytic activity of PMI|TiO_2|Pt. Conditions: 1.25 mg PMI|TiO_2|Pt, 3 mL TEOA buffer solution (0.1 M, pH 8.5), UV-filtered simulated solar light irradiation (100 mW cm^{-2}, AM 1.5G, λ > 420 nm) at 25 °C (see Table S7).

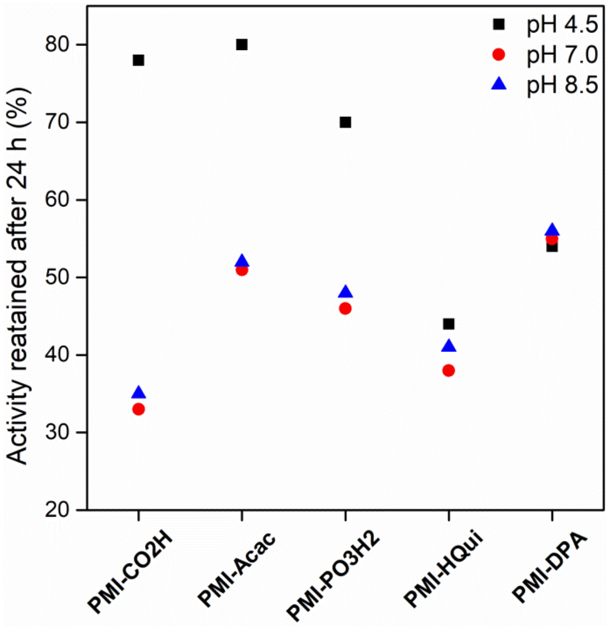

Figure S15. Comparison of the retained photocatalytic activity per pH and per PMI|TiO_2|Pt system after 24 h (data available in Table S5).

Supporting References

1. E. Bae and W. Choi, *J. Phys. Chem. B*, 2006, **110**, 14792.
2. M. A. Gross, A. Reynal, J. R. Durrant and E. Reisner, *J. Am. Chem. Soc.*, 2014, **136**, 356.
3. J. Fortage, M. Severac, C. Houarner-Rassin, Y. Pellegrin, E. Blart and F.Odobel, *J. Photochem. Photobiol. A* 2008*,* **197***, 156.*
4. V. A. Montes, R. Pohl, J. Shinar and P. Anzenbacher, *Chem.--Eur. J.,* 2006*,* ***12**, 4523.*
5. J. Boixel, E. Blart, Y. Pellegrin, F. Odobel, N. Perin, C. Chiorboli, S. Fracasso, M. Ravaglia, and F. Scandola *Chem. Eur. J.* 2010, **16**, 9140.
6. G. R. Fulmer, A. J. M. Miller, N. H. Sherden, H. E. Gottlieb, A. Nudelman, B. M. Stoltz, J. E. Bercaw and K. I. Goldberg, *Organometallics*, 2010, **29**, 2176.
7. D. T. Sawyer, J. L. Roberts, Jr. and A. Sobkowiak, *Electrochemistry for chemists*, New York (N.Y.) : Wiley, 2nd ed. edn., 1995.

End of Electronic Supporting Information
